# Non-viral *TRAC*-knocked-in CD19^KI^CAR-T and gp350^KI^CAR-T cells tested against Burkitt lymphomas with type 1 or 2 EBV infection: *In vivo* cellular dynamics and potency

**DOI:** 10.3389/fimmu.2023.1086433

**Published:** 2023-03-24

**Authors:** Tobias Braun, Alina Pruene, Milita Darguzyte, Alexander F. vom Stein, Phuong-Hien Nguyen, Dimitrios L. Wagner, Jonas Kath, Alicia Roig-Merino, Michael Heuser, Lucas L. Riehm, Andreas Schneider, Sabine Awerkiew, Steven R. Talbot, André Bleich, Constanca Figueiredo, Martin Bornhäuser, Renata Stripecke

**Affiliations:** ^1^ Clinic of Hematology, Hemostasis, Oncology and Stem Cell Transplantation, Hannover Medical School (MHH), Hannover, Germany; ^2^ University of Cologne, Faculty of Medicine and University Hospital Cologne, Department I of Internal Medicine, Center for Integrated Oncology Aachen Bonn Cologne Düsseldorf; Center for Molecular Medicine Cologne (CMMC), Cologne, Germany; ^3^ Institute for Translational Immune-Oncology, Cancer Research Center Cologne-Essen (CCCE), University of Cologne, Cologne, Germany; ^4^ Berlin Center for Advanced Therapies (BeCAT), Charité – Universitätsmedizin Berlin, Corporate Member of Freie Universität Berlin and Humboldt-Universität zu Berlin, Berlin, Germany; ^5^ BIH-Center for Regenerative Therapies (BCRT), Berlin Institute of Health (BIH) at Charité – Universitätsmedizin Berlin, Berlin, Germany; ^6^ Institute of Transfusion Medicine, Charité – Universitätsmedizin Berlin, Corporate Member of Freie Universität Berlin, Humboldt-Universität zu Berlin, Berlin, Germany; ^7^ MaxCyte Inc., Rockville, MD, United States; ^8^ Institute of Virology, Faculty of Medicine, University Hospital Cologne, University of Cologne, Cologne, Germany; ^9^ Institute for Laboratory Animal Science, MHH, Hannover, Germany; ^10^ Institute for Transfusion Medicine and Organ Engineering, MHH, Hannover, Germany; ^11^ Department of Internal Medicine 1, University Hospital Carl Gustav Carus, Technische Universität Dresden, Dresden, Germany; ^12^ German Center for Infection Research (DZIF), Partner site Hannover-Braunschweig, Hannover, Germany; ^13^ German Center for Infection Research (DZIF), Partner Site Bonn-Cologne, Cologne, Germany

**Keywords:** CAR-T cell, gene editing, CRISPR-Cas, lymphoma, Burkitt lymphoma, EBV, off-the-shelf, xenograft model

## Abstract

**Introduction:**

The ubiquitous Epstein–Barr virus (EBV) is an oncogenic herpes virus associated with several human malignancies. EBV is an immune-evasive pathogen that promotes CD8^+^ T cell exhaustion and dysregulates CD4^+^ T cell functions. Burkitt lymphoma (BL) is frequently associated with EBV infections. Since BL relapses after conventional therapies are difficult to treat, we evaluated prospective off-the-shelf edited CAR-T cell therapies targeting CD19 or the EBV gp350 cell surface antigen.

**Methods:**

We used CRISPR/Cas9 gene editing methods to knock in (KI) the CD19CAR.CD28z or gp350CAR.CD28z into the T cell receptor (TCR) alpha chain (*TRAC*) locus.

**Results:**

Applying upscaled methods with the ExPERT ATx^®^ MaxCyte system, KI efficacy was ~20% of the total ~2 × 10^8^ TCR-knocked-out (KO) generated cells. ^KO^TCR^KI^CAR-T cells were co-cultured *in vitro* with the gp350^+^CD19^+^ BL cell lines Daudi (infected with type 1 EBV) or with Jiyoye (harboring a lytic type 2 EBV). Both types of CAR-T cells showed cytotoxic effects against the BL lines *in vitro*. CD8^+ KI^CAR-T cells showed higher persistency than CD4^+ KI^CAR-T cells after *in vitro* co-culture with BL and upregulation of the activation/exhaustion markers PD-1, LAG-3, and TIM-3. Two preclinical *in vivo* xenograft models were set up with Nod.Rag.Gamma mice injected intravenously (i.v.) with 2 × 10^5^ Daudi/fLuc-GFP or with Jiyoye/fLuc-GFP cells. Compared with the non-treated controls, mice challenged with BL and treated with CD19^KI^CAR-T cells showed delayed lymphoma dissemination with lower EBV DNA load. Notably, for the Jiyoye/fLuc-GFP model, almost exclusively CD4^+^ CD19^KI^CAR-T cells were detectable at the endpoint analyses in the bone marrow, with increased frequencies of regulatory T cells (T_regs_) and TIM-3^+^CD4^+^ T cells. Administration of gp350^KI^CAR-T cells to mice after Jiyoye/GFP-fLuc challenge did not inhibit BL growth *in vivo* but reduced the EBV DNA load in the bone marrow and promoted gp350 antigen escape. CD8^+^PD-1^+^LAG-3^+^ gp350^KI^CAR-T cells were predominant in the bone marrow.

**Discussion:**

The two types of ^KO^TCR^KI^CAR-T cells showed different therapeutic effects and *in vivo* dynamics. These findings reflect the complexities of the immune escape mechanisms of EBV, which may interfere with the CAR-T cell property and potency and should be taken into account for future clinical translation.

## Introduction

Burkitt lymphoma (BL) and its leukemic manifestation Burkitt leukemia (B-AL) account for ~50% of non-Hodgkin lymphoma (NHL) in children and adolescents and is observed in ~1% of adult NHL cases in Europe and Northern America (1, 2). The 5-year event-free survival (EFS) rate for children and adolescents treated with anti-CD20 immunotherapy combined with chemotherapy [cyclophosphamide, doxorubicin, vincristine, and prednisolone (CHOP)] is >90% (1, 2). However, patients with relapsed BL have a poor chance of survival, and their 3-year EFS rate is less than 20% (3). BL is a malignancy of mature B cells expressing the antigens CD19, CD20, and CD22 along with monotypic surface immunoglobulin light chains (1, 4). Translocations involving the MYC oncogene and the immunoglobulin heavy chain (IGH) are observed in 80% of BL cases (2). Endemic BL is highly prevalent in Africa, and affected patients generally show Epstein–Barr virus (EBV) genomes detectable in tumor cells (5). Endemic EBV^+^ BL is mostly restricted to regions with a high incidence of malaria and human immunodeficiency virus (HIV) infections. In addition, sporadic and immunodeficiency-associated variants also exist. The full role of EBV in sporadic BL pathogenesis has not been completely elucidated, but EBV increases the chance that MYC-activating translocations, which drive lymphoma development, will be generated. Further, EBV latency has been implicated with pro-proliferative and anti-apoptotic functions in malignancies (6). Incidentally, recent findings showed that cellular transformation is not required for EBV maintenance, and it is the lytic cycle that mostly supports EBV-driven malignancies (6). Further, the EBV status of the sporadic BL subgroup increases with patient age and shows distinct pathogenic features similar to EBV^+^ endemic BL (7). This supports the general notion that EBV^+^ BL is an opportunistic malignancy that develops in immunocompromised patients unable to control viral reactivations. Thus, cell-based immunotherapy could be a good clinical approach to halt lytic EBV. EBV-specific T cells have been used successfully to treat post-transplant lymphoproliferative disease (PTLD) in immunocompromised patients following hematopoietic stem cell transplantation (HSCT) (8, 9), but this is not yet established for BL. Furthermore, genetically engineered T cells that could target EBV or cellular antigens have not yet been systematically evaluated for therapeutic uses against relapsed EBV^+^ lymphomas such as BL. To date, most chimeric antigen receptor (CAR)-T cell products approved by the United States Food and Drug Administration (FDA) and by the European Medicines Agency (EMA) for second-line immunotherapy in lymphomas and/or leukemias target the B-cell antigen CD19. The currently marketed CD19 CAR-T cell products are CTL019 (10), KTE-C19 (11), brexucabtagene autoleucel (12), and liso-cell (13). Generation of these CAR-T cells relies heavily on the transduction of T cells with lentiviral vectors (LVs) or retroviral vectors (RVs), which are costly and impose biosafety and gene technology level 2 restrictions (14). As an alternative to lymphoma antigens such as CD19 that are also expressed on normal B cells, resulting in their depletion, we explored viral antigens expressed on lymphoma cells for CAR engineering. The lytic EBV gp350 glycoprotein (encoded by BLLF1) is readily detectable on the surface of cells upon EBV lytic reactivation, and its expression persists in subsets of EBV^+^ latently infected cells (15). CAR-T cells generated after retroviral gene transfer and targeting gp350 have shown cytotoxicity *in vitro* against gp350^+^ EBV^+^ B-cell lines and *in vivo* in fully humanized mice infected with EBV and with recapitulating human PTLD and monomorphic diffuse large B-cell lymphoma (DLBCL) (15).

Here, we explored CAR-T cells targeting either CD19 or EBV/gp350 generated *via* non-viral gene transfer. Clustered regularly interspaced short palindromic repeat (CRISPR)-associated (Cas) 9 gene editing technology is an emerging non-viral “knock-in” (KI) approach for site-specific insertion of exogenous T cell receptors (TCRs) (16) or CARs (14). In this process, the TCR/CAR is inserted *via* homologous recombination within predetermined gene *loci* containing open reading frame sequences. In this way, the TCR/CAR insertion results in a knock-out (KO). Pioneering studies by Eyquem et al. have combined transfection of T cells with Cas9–single guide (sg)RNA ribonucleoprotein (RNP) complexes with transduction with a recombinant adeno-associated virus serotype 6 (rAAV6) to deliver the DNA donor template and homology-directed DNA repair (HDR) arms. The authors demonstrated the targeting of CAR integration into the first exon of the TCR-α constant gene (*TRAC*) (17). With the resulting TCR KO, *TRAC*-replaced CAR-T cells are highly specific against the target antigen and are promising candidates as allogenic cell therapy, since graft-versus-host-disease risks are potentially lower (18). Currently, non-viral CRISPR/Cas delivery approaches for TCR/CAR engineering are rapidly evolving (16, 19, 20). Most groups have used electroporation as a means of introducing the CAS enzyme, the gRNA ribonucleoproteins (RNPs), and the DNA HDR template (HDRT) into the cell (16, 21). With different optimization steps, Kath et al. demonstrated up to 50% efficacy of TCR^KO^CD19^KI^CAR-T cell generation (20). We have also demonstrated the potency of CD19^KI^CAR-T cells *in vivo* against the acute lymphoblastic leukemia (ALL) Nalm-6 cell line xenograft model (20). In this current work, we used the ExPERT ATx^®^ electroporation system to generate CD19^KI^CAR-T and gp350^KI^CAR-T cells. We tested the potency of the CAR-T cells against BL recapitulating latent (Daudi) and lytic (Jiyoye) EBV infections. Therapeutic use of CD19^KI^CAR-T cells significantly reduced BL spread for both *in vivo* models. Administration of gp350^KI^CAR-T cells lowered the EBV DNA load, but this did not produce *in vivo* therapeutic effects. Taken as a whole, the results provide proof-of-concept that EBV^+^ BL can be controlled with gene-edited TCR^KO^CAR^KI^-T cells. Future improvements in performance and facilitation of manufacturing can enable the development of an off-the-shelf product for rapid distribution to clinical centers to treat relapsed EBV^+^ BL patients.

## Material and methods

More information on the materials and methods can be found in the Supplementary Material. Reagents used for flow cytometry analyses are listed in **Table S1**.

### Ethics statement

Leukapheresis units were obtained under written informed consent of the donors in accordance with study protocols approved by Hannover Medical School Ethics Review Board (approval no. 4837). All experiments involving mice were performed in accordance with the German Animal Welfare Act and were approved by the Lower Saxony Office for Consumer Protection and Food Safety (“Niedersächsisches Landesamt für Verbraucherschutz und Lebensmittelsicherheit”, Dezernat 33/Tierschutz, LAVES; Protocol Nos. 33.12-42502-04-21/3791 and 33.12-42502-04-16/2347). Euthanasia was performed *via* cervical dislocation after CO_2_ inhalation.

### Cell lines

The HEK-293T cell line (abbreviated to 293T) was obtained from the American Tissue Culture Collection (CRL-11268™ ATCC, ATCC, Manassas, VA, USA). The lentivirally transduced 293T/gp350 cell line was previously established (15). 293T cells were cultured in Dulbecco’s Modified Eagle Medium (DMEM; Gibco™ by Thermo Fisher Scientific, Waltham, MA, USA) with 10% heat-inactivated fetal bovine serum (FBS; Gibco™), 100 units/ml penicillin G, and 100 µg/ml streptomycin sulfate (1× P/S; Sigma-Aldrich, St. Louis, MO, USA). Nalm-6 (ACC-128 https://www.dsmz.de/collection/catalogue/details/culture/ACC-128 German Collection of Microorganisms and Cell Cultures (DSMZ), Brunswick, Germany), Jiyoye (ACC590, https://www.dsmz.de/collection/catalogue/details/culture/ACC-590, DSMZ), and Daudi (ACC78, https://www.dsmz.de/collection/catalogue/details/culture/ACC-78, DSMZ) lymphoma cell lines were cultured in Roswell Park Memorial Institute (RPMI) (Gibco™) with 10% FBS and 1× P/S. All cell cultures were maintained at 37°C and 5% CO_2_. The cell lines were split every 2 to 3 days to maintain a density of 0.2–2.0 × 10^6^ cells/ml. Nalm-6, Daudi, and Jiyoye cell lines expressing fLuc-GFP were generated after transduction with the lentiviral vector pRRL.PPT.Cbx3-SFFV-fLuc-T2A-eGFP (the plasmid DNA containing the backbone vector was kindly provided by Prof. Axel Schambach, Institute of Experimental Hematology, Hannover Medical School, Germany). The cells were transduced with lentivirus for 24 h at 37°C and 5% CO_2_. At 72 h after transduction, the expression of green fluorescent protein (GFP) was confirmed by flow cytometry using the BD LSR II (BD Biosciences, Franklin Lakes, NJ, USA). The GFP^bright^ population was sorted by the Cell Sorting Core Facility at the Hannover Medical School. GFP^bright^ Daudi and Jiyoye cells were expanded in RPMI with 10% FBS and 1× P/S at 37°C and 5% CO_2_ and subsequently cryopreserved at −80°C.

### Primary cells

Apheresis collection was performed at the Institute of Transfusion Medicine and Organ Engineering (Hannover Medical School). Peripheral blood mononuclear cells (PBMCs) of Donors 1 and 3 were purified by sediment centrifugation (Ficoll, Biochrome AG, Berlin, Germany) and cryopreserved, whereas the cells of Donor 2 were not purified prior to cryopreservation. PBMCs were cultured in RPMI with 10% FBS and 1× P/S and supplemented with interleukin (IL)-7 (5 ng/ml) and IL-15 (5 ng/ml) (both from Miltenyi Biotec, Bergisch Gladbach, Germany). Media and cytokines were exchanged every 2 to 3 days.

### Design, amplification, and purification of HDRTs

**Figure 1:**
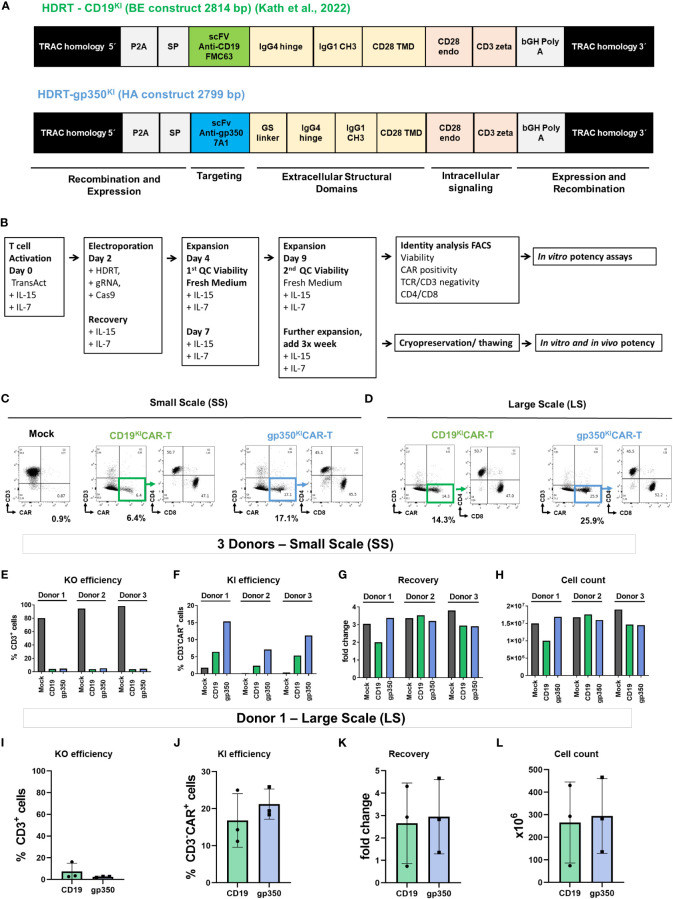
Generation of gene-edited TCR^KO^CAR^KI^-T cells. **(A)** Homology-directed DNA repair templates (HDRTs) containing the 5′ homology arms for the *TRAC* locus, a P2A element, a signal peptide (SP), the single-chain variable fragments (scFvs) for targeting the CAR against CD19 (derived from FMC63 mAb) or against gp350 (derived from 7A1 mAb), the GS linker, the IgG4 hinge, the IgG CH3 element, the CD28 transmembrane domain (TMD), the CD28 endocytoplasmatic domain, the CD3 zeta signaling domain, the bovine growth hormone (bGH) sequence for polyadenylation of the RNA transcript, and the 3′ homology arms for the *TRAC* locus. **(B)** Schematic representation of the main steps in gene editing: T cell activation, electroporation and recovery, homeostatic expansions, quality control analyses *via* flow cytometry, cryopreservation/thawing, and *in vitro* and *in vivo* potency tests. **(C, D)** Representative example of a small-scale (SS; C) or large-scale (LS; D) production of gene-edited CD19^KI^CAR-T and gp350^KI^CAR-T cells analyzed by flow cytometry (x-axis: CAR; y-axis: CD3). Relative to the mock T cells (not receiving the HDRT), the edited CAR-T cells showed loss of CD3 expression and gain of CAR expression. The CD3^−^CAR^+^ cells showed comparable frequencies of CD4^+^ and CD8^+^ cells. **(E–H)** Quantitative data for SC gene editing used with T cells obtained from three donors and analyzed on day 9 after initiation of cultures. **(E)** Reduced frequencies of CD3^+^ T cells as result of the *TRAC* knock-out (KO). **(F)** Frequencies of knock-in (KI) and expression of the CAR. **(G)** Recovery relative to cell input. **(H)** Final viable cell count on day 9. **(I–L)** Quantitative data for LS gene editing used as independent triplicates with T cells obtained from Donor 1 and analyzed on day 9 after initiation of cultures. **(I)** Reduced frequencies of CD3^+^ T cells as result of the *TRAC* KO. **(J)** Frequencies of KI and expression of the CAR. **(K)** Recovery relative to cell input. **(L)** Final viable cell count on day 9. Results for mock cells are depicted in black, CD19^KI^CAR-T cells in green, and gp350^KI^CAR-T cells in blue. The results represent cultures performed as independent triplicates not merged **(E–H)** or merged **(I–L)**.

The CD19CAR HDRT sequence has been previously described (20) (**Figures 1A**, **S1A**). The CD19CAR incorporates a single-chain variable fragment (scFv) derived from the anti-CD19 FMC63 antibody, similar to currently approved CD19CAR-T cell products. FMC63 is a mouse IgG2a monoclonal antibody with an equilibrium dissociation constant (*K*_D_) in the 10^−4^ nM range (22). The sequence of the CD19 HDRT is publicly available for academic and non-profit purposes (20) (see Addgene, https://www.addgene.org/183473/). The gp350CAR HDRT sequence was designed to incorporate the 7A1 scFv sequence targeting gp350. The parental 7A1 is a rat-derived monoclonal antibody, with high EBV *in vitro* neutralizing activity (15), but the *K_D_* was not determined (Stripecke et al., patent pending; further information and materials are available upon execution of a material transfer agreement with the Hannover Medical School). We developed and tested an HDRT named gp350CAR-BE with the same backbone structure as CD19CAR (20). In addition, we created the HDRT gp350CAR-HA with the same gp350CAR backbone previously validated in retroviral vectors (15) (**Figures 1A**, **S1A**). The plasmids containing the gp350CAR HDRT sequences were synthesized by Twist Bioscience (South San Francisco, CA, USA). The HDRT templates were amplified from the plasmids by PCR using Herculase II Fusion DNA Polymerase essentially as described (20) and following the vendor’s instructions (Agilent, Santa Clara, CA, USA). The PCR was performed with 3 ng plasmid in 50-µl reactions using primers homologous to the extremities of the TRAC homology arms (forward 5′-ataaaagaataagcagtattattaagtagccctgc-3′; reverse 5′-atctgcttttttcccgtgtcattct-3′). For amplification, the samples were placed in a PCR cycler and heated to 95°C for 2 min for initial denaturation; this was followed by 30 cycles of 20 s at 95°C, 20 s at 59°C, and 90 s at 72°C. A final step of extension at 72°C for 3 min was performed, before storage at 4°C until purification with DNA-binding magnetic beads (AMPure XP, Beckman Coulter, Brea, CA, USA). The beads were pre-warmed to room temperature (RT) for approximately 30 min, suspended by vortexing, and mixed one-to-one with up to 600 µl PCR product in a 1.5-ml tube. After 10 min of incubation at RT, the tube was placed on a DynaMag™-2 magnet (Thermo Fisher Scientific) for an additional 10 min at RT. The supernatant was removed while the tube was still on the magnet; 1.2 ml of 70% ethanol was added, and the sample was mixed by inverting the entire magnet. After 3 min of incubation, the ethanol was removed, and the washing step was repeated once. After the second wash, the pellet was air-dried for approximately 10 min to a semi-dry state. The tube was removed from the magnet, and the pellet was resuspended in 3 µl nuclease-free water per 100 µl used PCR product. After 2 min of incubation at RT, the tube was incubated for 5 more minutes on the magnet. The supernatants were collected without touching the bead fraction and diluted to 1:20 dilution in water, and the concentrations of the HDRTs were measured using a Nanophotometer^®^ (Implen, Munich, Germany). The DNAs were then adjusted to a concentration of 1 µg/µl with nuclease-free water and stored at −20°C until use.

### Generation of gene-edited CAR-T cells

PBMCs were activated with TransAct (Miltenyi Biotec) for 50–54 h using 10 µl of TransAct per 2 × 10^6^ cells per ml RPMI with 10% FBS and 1× P/S, and supplemented with IL-7 and IL-15 (5 ng/ml each). Cells were washed once with PBS and resuspended at 1 × 10^8^ cells/ml in TexMACS (Miltenyi Biotec). We used 50-µl cuvettes for small scale (SS) production (5 × 10^6^ cells per electroporation) and 1-ml cuvettes for large scale (LS) production (1 × 10^8^ cells per electroporation). To generate the RNP complex, 3 µM of crRNA TRAC guide 6 AGAGTCTCTCAGCTGGTACA (100 µM; IDT, Newark, NJ, USA) was mixed with 3 µM of tracrRNA (100 µM; IDT, catalog no. 1072532); the RNAs were diluted in a nuclease-free duplex buffer provided by IDT and kept cryopreserved in aliquots at −20°C. The RNAs were incubated at 95°C for 5 min to form the guide RNA. After being cooled to RT, 2.5 µg/ml 15–50 kDa poly-l-glutamic acid (PGA) (Sigma-Aldrich; 0.8:1 ratio PGA:gRNA) and 1 µM Cas9 (Alt-R S.p Cas9 Nuclease V3, 61 µM, IDT) were added, and the mixture incubated for 30 min to allow RNP formation. Subsequently, 50 µg/ml HDRT was added to the RNPs. R-50x8 processing assemblies (ER050U8-03, MaxCyte, Rockville, MD, USA) were used for SS and R-1000 processing assemblies (ER001M1-10, MaxCyte) for LS electroporation. An ExPERT ATx^®^ device (MaxCyte) was used for electroporation with the “Resting T cells 14.3” program. The electroporated cells were transferred into either a 96-well plate (Sarstedt, Nümbrecht, Germany; SS) or a 24-well plate (Sarstedt; LS) for recovery. The cuvettes were washed with 50 µl (R-50x8) or 1 ml (R-1000) TexMACS medium (Miltenyi Biotec) containing 2 µM HDR Enhancer V2 (IDT) and added to the corresponding wells, resulting in a final enhancer concentration of 1 µM. After 30 min recovery at 37°C, the cells were transferred into a 24-well plate (SS) or a T-75 flask (LS; Nunc-Thermo Fisher Scientific, Kamstrupvej, Denmark) containing the appropriate volume of pre-warmed RPMI to dilute the cell density to 2 × 10^6^ cells/ml. After 2 h at 37°C, cytokines (IL-7 and IL-15, each 5 ng/ml) and 10% FBS were added, and the cells were further cultured as described previously for T cells (the next medium exchange contained 1× P/S). An interim analysis was performed on day 4 after electroporation to confirm the KO (**Figure S1**). The time of analyses for KI was day 9 after electroporation, and for some experiments, cells were expanded for up to 14 or 19 days. Expanded cells were either freshly used or cryopreserved in freezing medium (20% FBS, 10% dimethyl sulfoxide (DMSO) in RPMI) and maintained at −150°C until being thawed for further use. For quality control, the gene-edited CAR-T cells were analyzed by flow cytometry for CD3-KO, CAR-KI, viability, and expression of CD4 and CD8 using an LSR II flow cytometer (BD). Cells were blocked in 100 µl PBS containing 10 µg/ml mouse IgG (Sigma-Aldrich). CAR KI was detected by staining with 1:50 Alexa Fluor^®^ 647 “AffiniPure F(ab’)_2_ Fragment Goat Anti-Human IgG” (109-606-170, Jackson Immuno Research Laboratories, West Grove, PA, USA). The staining of the CAR with the anti-human IgG was performed separately from the other subsequently used staining monoclonal antibodies (mAbs) to avoid complex formation. **Table S1** describes the antibodies used for staining. The cells were stained for 30 min on ice in darkness, washed twice in PBS containing 1% FBS, and resuspended in PBS. The data were acquired with an LSR II flow cytometer (BD Biosciences) and analyzed with FlowJo (Treestar).

### *In vitro* co-culture of CAR-T cells with target 293T cells and functional assays

The specificity and functionality of gp350^KI^CAR-T cells were tested through co-culture with 293T/WT or with 293T/gp350 cells. Target cells were seeded in a 96-well flat-bottom plate (1 × 10^4^ cells per well in 50 µl DMEM with 10% FBS and 1× P/S). The next day, we calculated the amount of effector ^KO^TCR^KI^CAR-T cells added to the culture at 1:1 and 3:1 effector to target (E:T) ratios. All cultures were performed in triplicate and were maintained at 37°C and 5% CO_2_ for 48 h. The supernatants of the triplicates were harvested and pooled, and IFN-γ secretion was analyzed using an ELISA kit in accordance with the manufacturer’s instructions (Human IFN-γ uncoated ELISA, Invitrogen, Carlsbad, CA, USA). Additionally, the concentrations of several human cytokines detectable in culture supernatants (GM-CSF, IFN-γ, IL-2, IL-5, IL-8, IL-10, IL-17A, IP-10, MCP-1, and TNF-α) were determined using a bead array Luminex kit in accordance with the manufacturer’s protocol (Milliplex Millipore, Burlington, MA, USA). The cells from the triplicate cultures were harvested, pooled, and washed with PBS, and the cell pellets were blocked in PBS containing 10 µg/ml mouse IgG (Sigma-Aldrich). To determine the number of viable cells, the cells were analyzed by flow cytometry (see **Figure S2**).

### *In vitro* co-culture of ^KI^CAR-T cells with target lymphoma cells and functional assays

For luminometry and ELISA analyses, Daudi/fLuc-GFP and Jiyoye/fLuc-GFP were seeded in 96-well round-bottom plates (1 × 10^4^ cells per well). ^KO^TCR^KI^CAR-T cell effectors were added to the culture at 1:1, 3:1, and 10:1 E:T ratios in 50 µl RPMI with 10% FBS and 1× P/S supplemented with IL-7 and IL-15 (both 5 ng/ml) per well. All co-cultures were performed in triplicate and were incubated for 72 h (at 37°C and 5% CO_2_). After incubation, the cells were spun down. The cell supernatants for each triplicate were collected for IFN-γ analyses using an ELISA kit (Human IFN-γ uncoated ELISA, Invitrogen, Waltham, MA, USA) in accordance with the manufacturer’s instructions. The cell pellets were resuspended in 100 µl d-luciferin potassium salt solution (SYNCHEM, Felsberg, Germany) (2.5 mg in 1 ml PBS). Luminescence emitted by living cells was measured for each instance with a TriStar2 instrument (Berthold Technologies, Bad Wildbad, Germany). The functionality of the large-scale cryopreserved/thawed CAR-T cells was also analyzed by flow cytometry. For this purpose, 1 × 10^6^ lymphoma target cells were seeded in a 12-well plate, and CAR-T cells were added at a 1:1 E:T ratio (adjusted to the CD3^−^CAR^+^ population) in 500 µl RPMI with 10% FBS and 1× P/S supplemented with IL-7 and IL-15 (both 5 ng/ml) per well. The co-cultures were incubated for 72 h (at 37°C and 5% CO_2_). The cells were harvested, washed with PBS, and spun down. The pellets were blocked in 100 µl PBS containing 10 µg/ml mouse IgG (Sigma-Aldrich). Cells were stained with 1:200 Fixable Viability Dye eFluor™ 450 (FVD450, 65-0863-14; Invitrogen) for viability control and with 1:400 PerCP anti-human CD4 (317432, BioLegend, San Diego, CA, USA) and 1:200 PE-Cy7 anti-human CD8 (300914, BioLegend). Viable cells (FVD450^−^), target cells (GFP^+^), and CD4^+^ or CD8^+^ CAR-T cells were identified by flow cytometry using the LSR II device (BD) (see **Table S1A** for antibodies and **Figure S3** for gating strategy details).

### Flow cytometry analyses of exhaustion markers and regulatory T cells

First, 1 × 10^6^ CAR-T cells in 1 ml R10 were seeded in a 6-well plate, to which 1 × 10^6^ GFP^+^ target cells in 1 ml R10 were added. IL-7 and IL-15 were added at 5 ng/ml each, and the plates were incubated at 37°C and 5% CO_2_. At each time point of the culturing process (24, 48, and 72 h), 0.4 ml of the cell suspension was taken for cell counting, staining, and flow cytometry analysis. For BM analyses, cryopreserved samples were thawed, maintained in culture for 2 h in R10, and analyzed. For staining of exhaustion markers, the cells were centrifuged at 300 g for 5 min and resuspended in PBS. Since anti-Fc CAR antibodies can potentially bind to other antibodies, the staining procedure was performed in two steps. First, the samples were blocked with mouse IgG (Sigma Aldrich) for 10 min on ice, and CAR staining was then performed for 30 min at RT. The cells were next washed with FACS buffer (PBS with 1% FCS) and centrifuged. Subsequently, the samples were resuspended in a master mix containing all other antibodies and stained for 30 min at RT (see **Table S1B** for antibodies and **Figure S6** for gating strategy details). Finally, cells were washed with FACS buffer, centrifuged, and resuspended in PBS. For staining of regulatory T cells (T_regs_), the samples were collected in FACS tubes, centrifuged at 300 g for 5 min, and resuspended in PBS. First, the samples were blocked with FcR (Miltenyi Biotec, cat. #130-059-901) for 10 min, and the cells were stained for 20 min at RT for detection of CD4. Intracellular labeling of FoxP3 was carried out using the IntraPrep Permeabilization Reagent kit (Beckman Coulter, cat. #A07803) in accordance with the manufacturer’s instructions (see **Table S1C** for antibodies and **Figure S7** for gating strategy details). All samples were washed twice with PBS and measured using a MACSQuant X Flow Cytometer, and the analyses were performed using FlowJo software (version 10.8).

### Establishment of *in vivo* xenograft models and bioluminescence imaging analyses

NOD.Cg-Rag1*^tm1Mom^* IL-2Rγc*^tm1Wjl^* (NRG) mice were obtained from the Jackson Laboratory (JAX, Bar Harbor, ME, USA) and bred under pathogen-free conditions or used for experiments. Mice aged 6–12 weeks were used in the present study. In experimental group mice, 2 × 10^5^ Daudi/GFP-fLuc or 2 × 10^5^ Jiyoye/GFP-fLuc cells were injected intravenously (i.v.)into the tail vein; control mice were injected with PBS. Bioluminescence imaging (BLI) analyses were performed with the IVIS Spectrum CT apparatus (PerkinElmer, Waltham, MA, USA), and the data were analyzed using LivingImage software (PerkinElmer) as previously described (20). Briefly, mice were anesthetized using isoflurane and injected intraperitoneally (i.p.) with 2.5 mg d-luciferin potassium salt dissolved in 100 µl PBS. The mice were imaged in frontal and lateral view with an automated exposure time of up to 5 min. Female mice showed higher rates of engraftment and tumor outgrowth (see **Figure S4**) and were thus used for testing CAR-T cells. The first imaging analysis was performed at 4 days after lymphoma challenge (D4) to confirm tumor engraftment, to determine the baseline for each mouse, and for allocation of mice to experimental groups along with others with similar engraftments. Additional sequential analyses were performed at 2, 3, and 3–4 weeks after lymphoma challenge. After euthanasia, macroscopic analyses were performed with the open abdomen and chest, and imaging analyses were performed post-mortem for direct visualization of tumor dissemination in organs. Samples of peripheral blood (PBL), bone marrow (BM), spleen (SPL), and lymph nodes (LNs) were collected for flow cytometry for characterization of GFP, CD20, CD19, and gp350 expression (gating strategy details are shown in **Figure S4**).

### *In vivo* potency testing of TCR^KO^CD19^KI^CAR-T and TCR^KO^gp350^KI^CAR-T cells

Lymphoma engraftment was determined by BLI on D4. Mice were distributed into cohorts with comparable engraftments, and 1 × 10^6 KO^TCR^KI^CAR-T cell effectors were injected i.v. During the experimental period of 3–4 weeks, the mice were monitored (depending on clinical presentation) daily or at least three times per week for weight, morbidity, and any clinical sign of graft-versus-host disease (GvHD). BLI analyses were performed weekly. The experiment was terminated if mice met the LAVES-approved euthanasia criteria (body weight reduction of ≥20%, ruffled fur, reduced activity, and/or self-isolation). After euthanasia, macroscopic examinations of the SPL, LNs, kidneys, and ovaries were performed, and the number of tumors was counted. The BM was flushed from both femora and pooled for each mouse. Some BM cells were snap-frozen for isolation of DNA, some were used for BM smears, and the rest were used for flow cytometry analysis or cryopreserved. The LNs and SPL were smashed through a 100-µm cell strainer to obtain single-cell suspensions. SPL and PBL were resuspended in erythrolysis buffer (0.83% ammonium chloride, 20 mM HEPES [pH 7.2]), incubated for 5 min at RT, and then washed with PBS. The number of viable cells in each tissue sample was quantified by Trypan blue exclusion. The cells were analyzed fresh or cryopreserved in freezing medium (20% FBS and 10% DMSO in RPMI) and maintained at −150°C.

### Flow cytometry analyses of cells recovered from mouse tissues

Two panels were used for flow cytometry analyses: 1) to quantify the GFP^+^ lymphoma load and determine the expression of CD19 and gp350 antigens on the surface of GFP^+^CD45^+^CD20^+^ lymphoma cells (7A1 mAb + secondary Ab; or, to determine the background, secondary Ab only) and 2) to identify and quantify CAR-T cells (CD45^+^GFP^−^, CD4, CD8, and CAR). The list of antibodies used and amounts used per staining are shown in **Table S1A**, and gating strategies are shown in **Figures S4, 5**. Approximately 5 × 10^5^ cells (lymphocytes isolated from mice, *in vitro* cultured tumor cell lines, or gene-edited T cells) were blocked for 30 min on ice in 100 µl of 10 µg/µl mouse IgG (Sigma-Aldrich) in PBS and then washed with 1% FBS in PBS. Analyses were performed using a BD LSR II flow cytometer.

### Microscopic analyses of BM smears

The Giemsa staining method used for BM smears is described in the **Supplementary Material**. The morphology of bone marrow was assessed with an Olympus BX51 (Olympus, Tokyo, Japan) microscope and a 40×/0.75 numerical aperture objective or a 100×/1.3 numerical aperture objective with Zeiss Immersol Medium (Zeiss, Jena, Germany). OlympusXC50 (Olympus) and analySIS software (Soft Imaging System, Stuttgart, Germany) were used to capture images.

### Quantification of EBV copies in BM

DNA isolation of mouse BM to determine the EBV load was performed with a QIAamp DNA Blood Mini Kit (Qiagen, Valencia, CA, USA) according to the manufacturer’s protocol. DNA concentration and purity were measured using the Nanodrop instrument. The DNA samples showed similar concentrations, and 10 μl was used per reaction. The EBV DNA detection by real-time PCR was performed with the EBV *in vitro* diagnostics PCR kit (GeneProof^®^, Dolni Herspice, Brno, Czechia) after amplification of a specific conserved DNA sequence of the single-copy gene encoding the nuclear antigen 1 (EBNA1) and measurement of fluorescence emission. An internal standard (IS) was included in the reaction mix as a control for the PCR reaction. The detection kit explores the “hot start” technology, minimizing non-specific reactions and assuring maximum sensitivity. The analyses were performed using a LightCycler 2.0/480 instrument. The detection limit of the assay was approximately 1.9 × 10^5^ EBV units in the sample.

### Statistical analysis

For data obtained in *in vitro* killing assays, t-tests were used to compare the experimental groups with controls. For *in vivo* data, the analyses were performed by a biostatistician (S. R. Talbot, co-author), and some datasets were analyzed using ANOVA or Kruskal–Wallis (in either case, with Tukey’s post-hoc testing to compare the three groups), or *via* a binomial regression, which was used for statistical analyses and group comparisons. Additional methods and references for statistical analysis are described in the **Supplementary Material**. The significance level was set to 0.05. Statistical analyses were carried out using GraphPad Prism V7.0 software (GraphPad Software, La Jolla, CA, USA) and R software V4.1.0 (R Foundation for Statistical Computing, Vienna, Austria).

## Results

### Validation of an HDRT for redirecting gene-edited CAR-T cells against EBV/gp350 and adaptation of methods using MaxCyte electroporation systems for the small and large scale

In a previous study, Kath et al. presented technical improvements for fast and efficient virus-free insertion of CARs into the *TRAC* locus of T cells (20). The HDRT used for generation of CD19-specific gene-edited CARs contained a CD28 costimulatory domain, followed by the CD3 zeta effector chain (**Figure 1A**; see additional elements explained in legends). Here, we designed and tested two HDRTs for redirecting the CAR-T cells against the EBV/gp350 protein (**Figures 1A**, **S1A**). We compared a gp350CAR-BE HDRT containing the CD19CAR backbone with a gp350CAR-HA HDRT, analogous to a backbone previously validated in a retroviral vector (15). The gp350CAR-HA HDRT incorporating additional linkers and a different membrane export signal resulted in higher CAR expression (**Figure S1**); therefore, it was used in further experiments. For the current set of studies, we adapted the cell processing steps (**Figure 1B**) in terms of upscaling capability with the ExPERT ATx^®^ system. This electroporation system is modular and can be developed from the preclinical small scale (SS, for *in vitro* testing) and preclinical large scale (LS, for *in vitro* and *in vivo* testing) toward an up-scaled clinical scale (for use in patients). The gene-edited cells were harvested after expansion, analyzed for identity, and tested *in vitro* or *in vivo*. SS productions with T cells obtained from three different donors were performed in small electroporation cuvettes (50 µl, 5 × 10^6^ cells; **Figure 1C**, see gating strategy used for flow cytometry analyses in **Figures S1B, C**). LS productions were generated with T cells from a single donor (Donor 1) and were performed in larger electroporation cuvettes (1 ml, 1 × 10^8^ cells; **Figure 1D**). For both the SS and LS methods, the knock-outs of the TCRα chain resulted in major reductions in CD3 detection on the cell surface (**Figures 1E, I**). Within the CD3^−^ population, CAR KI was variable within donors used for the SS and within CARs (~3%–5% for CD19^KI^CAR and ~10%–15% for gp350^KI^CAR; **Figure 1F**). The PBMCs obtained from Donors 1 and 3, which were purified by Ficoll sediment centrifugation and used as a source of T cells, showed a better KI than the PBMCs from Donor 2, which were not purified and showed lower cell viability. KI efficiency was higher for the LS runs and comparable for both CARs (~20%, **Figure 1J**). At day 9 of cell culture, T cell recovery was increased on average threefold relative to input for SS and LS (**Figures 1G, K**), resulting in total yields of ~1.5 × 10^7^ cells for SS (**Figure 1H**) and ~3.5 × 10^8^ total cells for LS (**Figure 1L**). After these key adaptation steps, the functional properties of ^KI^CAR-T cells were analyzed.

### Gene-edited TCR^KO^gp350^KI^CAR-T cells recognized gp350 specifically on target cells

**Figure 2:**
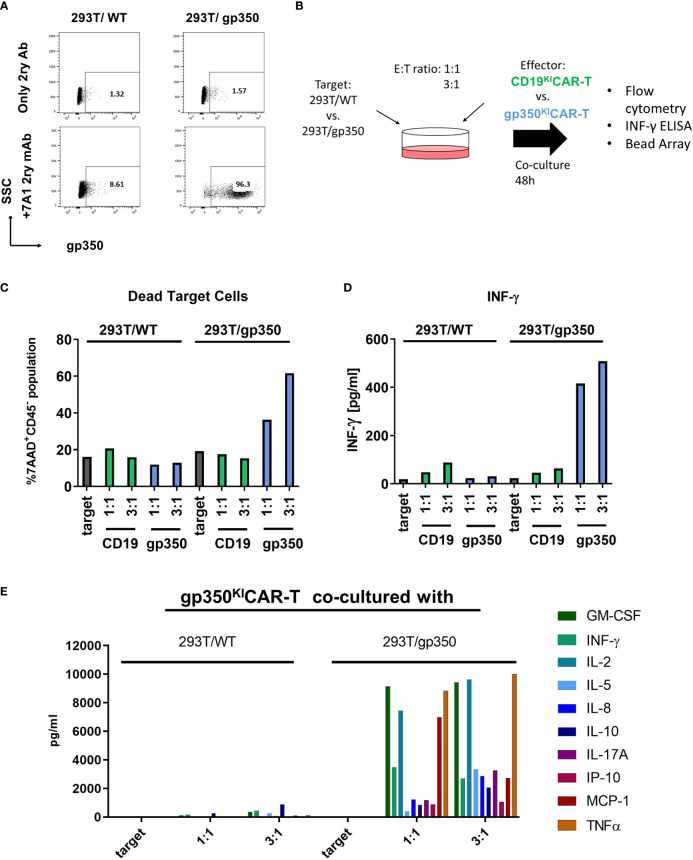
Specific recognition of gp350 on the cell surface by gp350^KI^CAR-T cells. **(A)** Flow cytometry analyses of gp350 expression on the cell surface of 293T/WT and 293T/gp350 cell lines. Upper panels show cells stained only with the immune-conjugated secondary antibody. Lower panels show cells stained with the primary 7A1 monoclonal antibody and the secondary antibody. **(B)** Schematic representation of analyses of gp350^KI^CAR-T cell specificity after co-culture with 293T/WT or 293T/gp350 cell lines at different effector-to-target (E:T) ratios. After 48 h of co-culture, the dead cells were detected by flow cytometry, and cytokines secreted in the medium supernatant were analyzed. **(C)** Quantified proportions of dead target cells. **(D)** Secreted IFN-γ (pg/ml) and **(E)** several cytokines detected in cell supernatants (pg/ml) after co-cultures at 1:1 and 3:1 E:T ratios. Data obtained with CD19^KI^CAR-T cells with gp350^KI^CAR-T cells are depicted in green and blue, respectively. The results represent a single assay.

As a first *in vitro* analysis of the cytotoxic potency and specificity of gp350^KI^CAR-T cells, we used a 293T cell line with stable expression of gp350 (15) (**Figure 2A**). Co-cultures of SC CD19^KI^CAR-T or gp350^KI^CAR-T with 293T/WT or 293T/gp350 as target cells were performed with E:T ratios of 1:1 and 3:1 for 48 h (**Figure 2B**). The co-cultures were then stained for detection of CD45 (a pan-hematopoietic marker not expressed on 293T cells) and 7AAD to determine the fractions of dead and viable cells (see gating strategy details in **Figure S2**). Co-culturing of CAR-T cells against either CD19 or against gp350 with 293T/WT cells resulted in a proportion of dead target cells of approximately 20%, which was comparable with the proportion of dead cells in the culture of target cells without CAR-T cells (**Figure 2C**). Co-culture of 293T/gp350 with gp350^KI^CAR-T cells showed 40%–60% killing, whereas the other co-cultures showed approximately 20% background loss of survival. Supernatants were harvested, and the secreted cytokines were quantified by ELISA or bead array. Only the co-culture of gp350^KI^CAR-T cells with 293T/gp350 showed elevated levels of IFN-γ, GM-CSF, IL-2, IL-5, IL-8, IL-10, IL-17A, MCP-1, and TNF-α secretion (**Figures 2D, E**). These results confirmed the specificity of gp350^KI^CAR-T cells and indicated an E:T dose-dependent effect for killing upon exposure to gp350^+^ cell targets.

### Gene-edited ^KI^CAR-T cells produced on a small scale recognized and killed EBV^+^ Burkitt lymphoma cell lines *in vitro*

**Figure 3:**
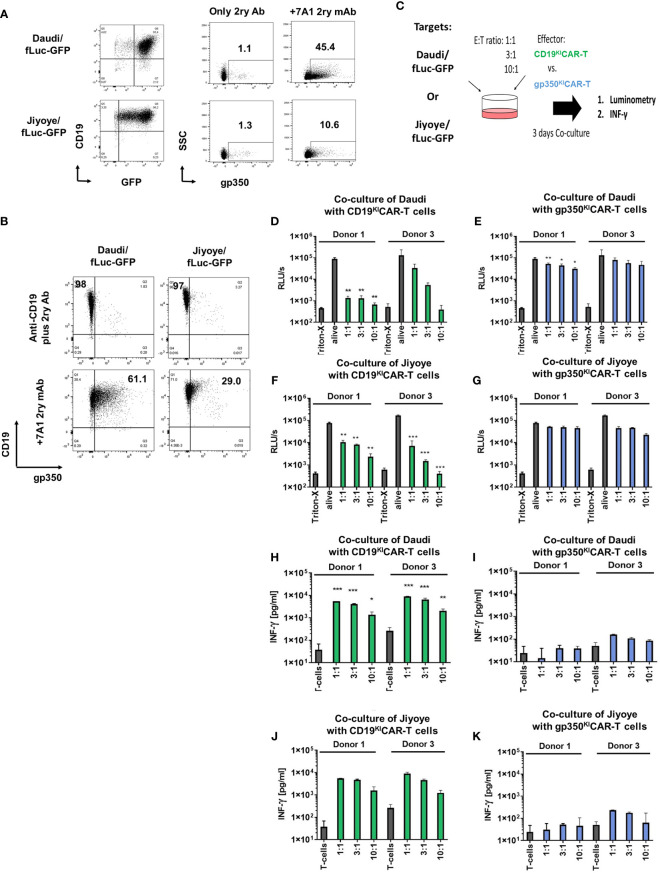
CD19^KI^CAR-T and gp350^KI^CAR-T generated on a small scale recognized and killed Daudi/fLuc-GFP (EBV^+^ type 1) and Jiyoye/fLuc-GFP (EBV^+^ type 2) cell lines *in vitro*. **(A)** Left panels: flow cytometry analyses of Daudi/fLuc-GFP and Jiyoye/fLuc-GFP cell lines showing GFP^+^CD19^+^ cells. Right panels: specific expression of gp350 after staining with the anti-gp350 7A1 antibody, followed by staining with a conjugated secondary antibody, is shown for each target cell line. **(B)** More than 97% of the Daudi/fLuc-GFP and Jiyoye/fLuc-GFP cell lines express high levels of CD19, whereas within the CD19-positive cells, only subpopulations of Daudi/fLuc-GFP (61%) and Jiyoye/fLuc-GFP (29%) express variable levels of gp350. **(C)** Schematic representation for functional analyses of CD19^KI^CAR-T and gp350^KI^CAR-T cells after co-culture with Daudi/fLuc-GFP or with Jiyoye/fLuc-GFP cell lines at different E:T ratios. After 3 days of co-culture, cells were analyzed by luminometry (relative light units per second; RLU/s) and IFN-γ secretion. Triton-X was used to determine the maximum killing rate, whereas non-treated alive cells were used as controls. **(D, E)** Cytotoxicity effects of CD19^KI^CAR-T or gp350^KI^CAR-T effectors after co-culture with Daudi/fLuc-GFP targets. **(F, G)** Cytotoxicity effects of CD19^KI^CAR-T or gp350^KI^CAR-T effectors after co-culture with Jiyoye/fLuc-GFP targets. **(H, I)** IFN-γ detected in cultures of CD19^KI^CAR-T or gp350^KI^CAR-T effectors after co-culture with Daudi/fLuc-GFP targets. **(J, K)** IFN-γ detected in cultures of CD19^KI^CAR-T or gp350^KI^CAR-T effectors after co-culture with Jiyoye/fLuc-GFP targets. Results for CD19^KI^CAR-T cells are depicted in green and those for gp350^KI^CAR-T cells in blue. The results represent cultures performed in triplicate. Statistical analyses took the form of a one-tailed grouped t-test (***p < 0.001, **p < 0.01, *p < 0.05).

As the next step, we evaluated the capacity of CD19^KI^CAR-T and gp350^KI^CAR-T cells to react *in vitro* against BL cells infected with EBV. Humans can be infected with two types of EBV: type 1 (T1) and type 2 (T2). T1 EBV transforms B cells more efficiently than T2 EBV *in vitro*, and T2 EBV-infected B cells can produce more lytic virus. As putative cell line models derived from pediatric BL patients, we obtained commercially CD19^+^CD20^+^EBV^+^ human B-lymphoblastoid T1 Daudi (23) and T2 Jiyoye cells (24). Both cell lines are considered prototypic mature B cells and, as such, express costimulatory ligands such as CD80 and CD86. The Daudi cell line is positive for EBV/EBNA by PCR analyses and expresses the mRNA for the proto-oncogene BCL-2, but the BLZF-1 immediate-early protein and capsid protein had previously not been detectable by Western blotting after phorbol ester/sodium butyrate stimulation. Although EBV infection in Daudi was classified as latent, Daudi cells express glucocorticoid receptors (GRs) and show a glucocorticoid dose-dependent upregulation of BZLF1 mRNA expression by hydrocortisone (HC) and dexamethasone (Dex). Dex induces expression of the early gene products BLLF3 (encoding for the EBV dUTPase) and BALF5 (encoding for the EBV DNA polymerase) and thus can promote EBV reactivations (25). The Jiyoye cell line is positive for EBV/EBNA by PCR analyses, carries the t(8:14) translocation rearranging IGH with MYC, and expresses the BLZF-1 immediate-early protein after phorbol ester/sodium butyrate stimulation and Western blot analyses. The EBV infection in Jiyoye was classified as potentially lytic with the possibility of production of active virus. In order to set up *in vitro* and *in vivo* assays, Daudi and Jiyoye cell lines co-expressing fLuc and GFP were generated using lentiviral transduction. Co-expression of CD19 and the reporter GFP was confirmed by flow cytometry (**Figure 3A**, left panels). When gated on the GFP^+^ cells, approximately 45% of the Daudi/fLuc-GFP cells and 10% of the Jiyoye/fLuc-GFP cells expressed gp350 (**Figure 3A**, right panels). Within the bright and homogeneously expressing CD19^+^ population (corresponding to >95% of the BL cells), 61% of the Daudi/fLuc-GFP and 29% of the Jiyoye/fLuc-GFP expressed gp350, but at much lower and variable levels (**Figure 3B**). It is known that EBV reactivation can be induced in BL cells, for example by treatment with phorbol esters (e.g., phorbol myristate acetate [PMA]). One explanation for the variable and relatively low levels of gp350 expression is that cells displaying very high lytic reactivation might be more prone to modulations of cell cycle control, although the specific functions of gp350 as an apoptotic or anti-apoptotic factor remain to be fully determined (26). Both target cell lines were cultured for 3 days with CD19^KI^CAR-T cells or gp350^KI^CAR-T cells in different E:T ratios (1:1, 3:1, and 10:1) (**Figure 3C**). The *in vitro* assay to test the CAR-T cells utilized fLuc as a vitality reporter gene followed by luminometry, as viable cells produce high levels of bioluminescence while dead cells do not (**Figure 3C**). In addition, T cell activation was followed by measurement of IFN-γ secreted in the supernatant (**Figure 3C**). CAR-T cells produced as SS from Donors 1 and 3 were used for co-culturing in order to evaluate donor-dependent differences. CD19^KI^CAR-T cells produced from both donors greatly reduced the number of living Daudi/fLuc-GFP and Jiyoye/fLuc-GFP cells after 3 days of co-culture at all tested E:T ratios (1:1, 3:1, and 10:1) (**Figures 3D, F**). This was associated with a dose dependent secretion of INF-γ detectable in supernatants (**Figures 3H, J**). The co-culture gp350^KI^CAR-T cells with Daudi/fLuc-GFP or Jiyoye/fLuc-GFP target cells stimulated modest but dose-dependent cytotoxic effects (**Figures 3E, G**) and detectable but low levels of INF-γ (**Figures 3I, K**). In sum, SS CD19^KI^CAR-T cells showed robust cytotoxic effects against both types of lymphomas, while the functional effects of gp350^KI^CAR-T cells were low.

### ^KI^CAR-T cells produced at LS, cryopreserved, and thawed showed higher *in vitro* potency

**Figure 4:**
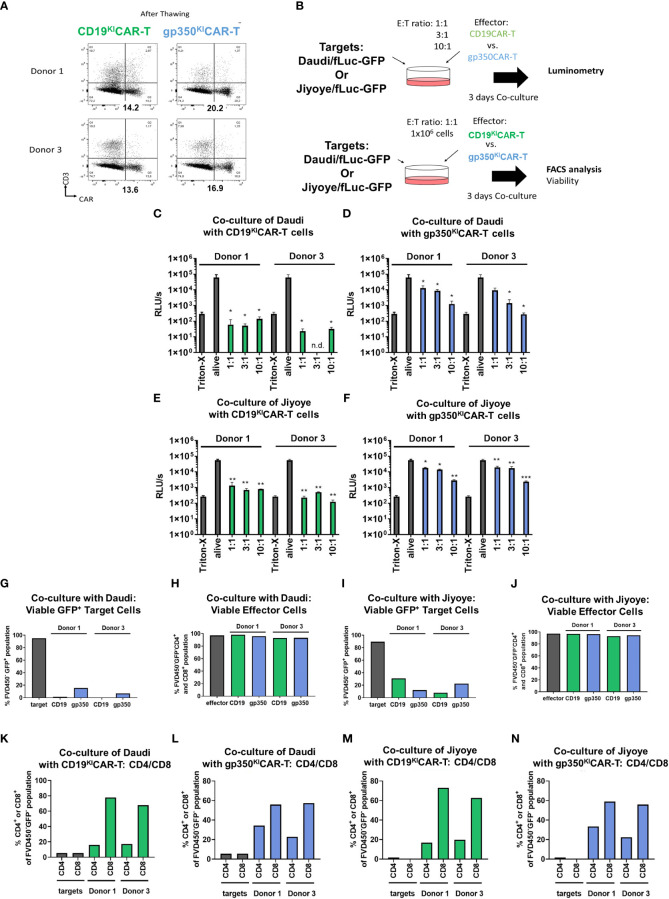
CAR^KI^-T cells produced on a large scale, cryopreserved and thawed, show improved killing of EBV^+^ Burkitt lymphoma cell lines *in vitro*. **(A)** Flow cytometry analyses (x-axis: CAR; y-axis: CD3) of CD19^KI^CAR-T and gp350^KI^CAR-T cells generated with PBMCs of two healthy donors after cryopreservation and thawing. **(B)** Schematic representation of functional analyses of CD19^KI^CAR-T and gp350^KI^CAR-T cells after co-culture with Daudi/fLuc-GFP and Jiyoye/fLuc-GFP cell lines at different E:T ratios. After 3 days of co-culture, cell killing was quantified *via* luminometry (upper diagram) and flow cytometry (lower diagram). **(C–F)** Luminometry detection (relative light units per second (RLU/s)). Triton-X was used to determine the maximum rate of killing, whereas non-treated alive cells were used as controls. **(C, E)** CD19^KI^CAR-T effectors co-cultured with Daudi/fLuc-GFP or Jiyoye/fLuc-GFP targets. **(D, F)** gp350^KI^CAR-T effectors co-cultured with Daudi/fLuc-GFP or Jiyoye/fLuc-GFP targets. **(G, H)** Frequencies of Daudi/fLuc-GFP targets and effectors detected after co-culture. **(I, J)** Frequencies of Jiyoye/fLuc-GFP targets and effectors detected after co-culture. **(K, L)** Frequencies of CD4^+^ and CD8^+^ subpopulations after co-culture with Daudi/fLuc-GFP targets. **(M, N)** Frequencies of CD4^+^ and CD8^+^ subpopulations after co-culture with Jiyoye/fLuc-GFP targets. Results for CD19^KI^CAR-T cells are depicted in green and those for gp350^KI^CAR-T cells in blue. The luminometry results **(C–F)** represent cultures performed in triplicate; for flow cytometry, the samples were pooled **(K–N)**. Statistical analyses took the form of a one-tailed grouped t-test (***p < 0.001, **p < 0.01, *p < 0.05).

One important aspect in banking of ^KI^CAR-T cells is that they must remain unaffected by cryopreservation and thawing. Therefore, we further evaluated the properties and functionality of cryopreserved/thawed ^KI^CAR-T cells produced at LS from two different donors. CAR expression was observed in the range of 13%–20% after thawing (**Figure 4A**). The functionality was determined by two analytical assays: one using luminometry and the other based on flow cytometry (**Figure 4B**, see gating strategy details in **Figure S3**). The luminometry-based assays performed with Daudi/fLuc-GFP or with Jiyoye/fLuc-GFP target cells showed significant dose-dependent killing results for CD19^KI^CAR-T (**Figures 4C, E**) and gp350^KI^CAR-T (**Figures 4D, F**). CD19^KI^CAR-T cells were more efficient in killing than gp350^KI^CAR-T cells. The flow cytometry-based assay confirmed efficient killing for both types of ^KI^CAR-T cells, with high viability of the effector cells (**Figures 4G–J**). Furthermore, the effector cells remaining in the cultures could be distinguished by flow cytometry as CD4^+^ (T helper type, T_h_) and CD8^+^ (cytotoxic T lymphocyte (CTL) type; see gating strategy details in **Figure S3A**). For all *in vitro* cultures, higher frequencies of CTLs were observed (**Figures 4K–N**). In sum, these results showed better killing performance of large-scale ^KI^CART-T cells and dominance of CTLs *in vitro*.

### Evaluation of ^KI^CAR-T cells in the Daudi/fLuc-GFP xenograft model

**Figure 5:**
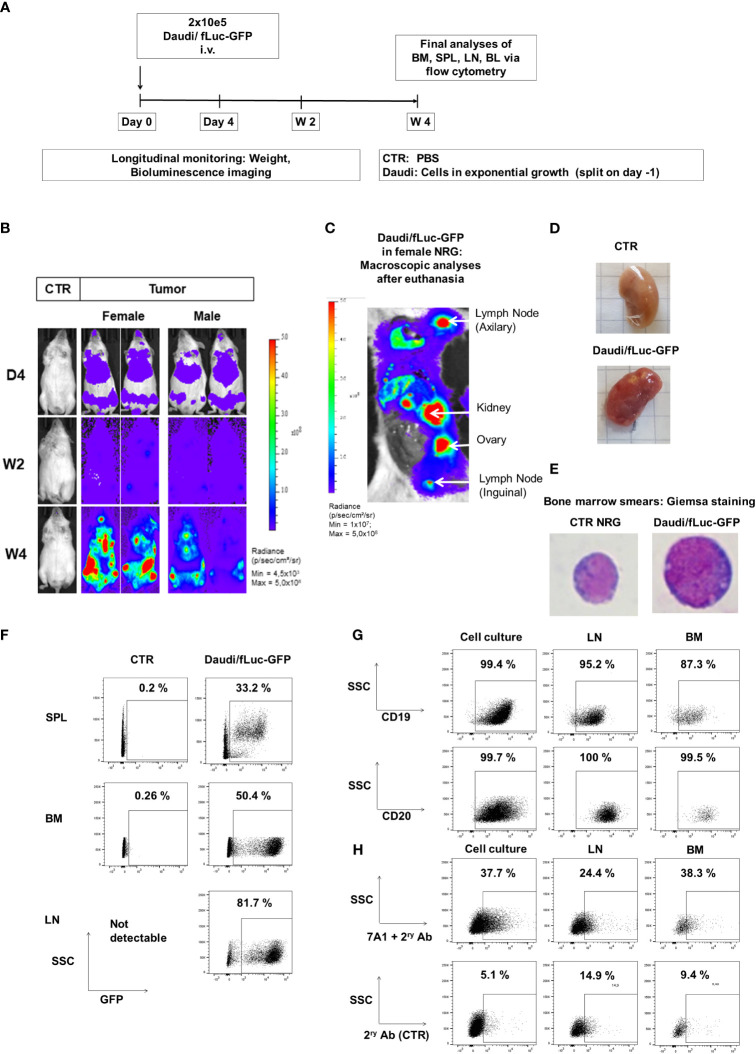
Daudi/fLuc-GFP engrafted and disseminated in several organs of mice and maintenance of surface expression of GFP, CD19, CD20, and gp350 *in vivo*. **(A)** Experimental scheme. Nod.Rag.Gamma (NRG) mice were challenged with 2 × 10^5^ Daudi/fLuc-GFP cells (i.v. injection; females, n = 3; males, n = 3; non-challenged PBS control, CTR, n = 1). **(B)** Representative examples of two challenged mice (female and male) and a control mouse are shown. Mice were analyzed longitudinally by bioluminescence imaging (BLI) for 4 weeks, and the experiment was then terminated. BLI of control (PBS) and female and male mice is shown at 4 days, 2 weeks, and 4 weeks after challenge (frontal view; scale from 4.5 × 10³ to 5.0 × 10^8^ p/s/cm^2^/sr). **(C)** Post-mortem abdominal BLI of a female mouse developing several lymphoma *foci* in lymph nodes, kidney, and ovary (scale 1.0 × 10^7^ to 5.0 × 10^8^ p/s/cm^2^/sr). **(D)** Images of kidneys, from CTR mouse as a reference and from a mouse developing tumors. **(E)** Images of bone marrow smears stained with Giemsa. A mouse normoblast was seen in control mice, and cells with typical morphology of lymphoma were observed in a mouse challenged with Daudi/fLuc-GFP cells. Original magnification ×1,000. **(F)** Flow cytometry analyses of various lymphatic tissues (SPL, spleen; BM, bone marrow; LNs, lymph nodes). GFP expression (shown in the x-axis) was maintained in blasts; 10,000 events are shown. **(G)** Flow cytometry analyses of Daudi/fLuc-GFP cells maintained in culture or explanted from LNs and BM. GFP^+^ blasts express CD19 and CD20. At least 1,500 events are shown. **(H)** Flow cytometry analyses of Daudi/fLuc-GFP cells maintained in culture or explanted from LNs and BM. Subpopulations of GFP^+^CD20^+^ blasts expressed gp350. The samples were stained with 7A1 primary mAb for gp350 detection and secondary Ab (upper row) or with secondary Ab only (bottom row). At least 1,500 events are shown. Unstained samples were used as references for gating (not shown).

**Figure 6:**
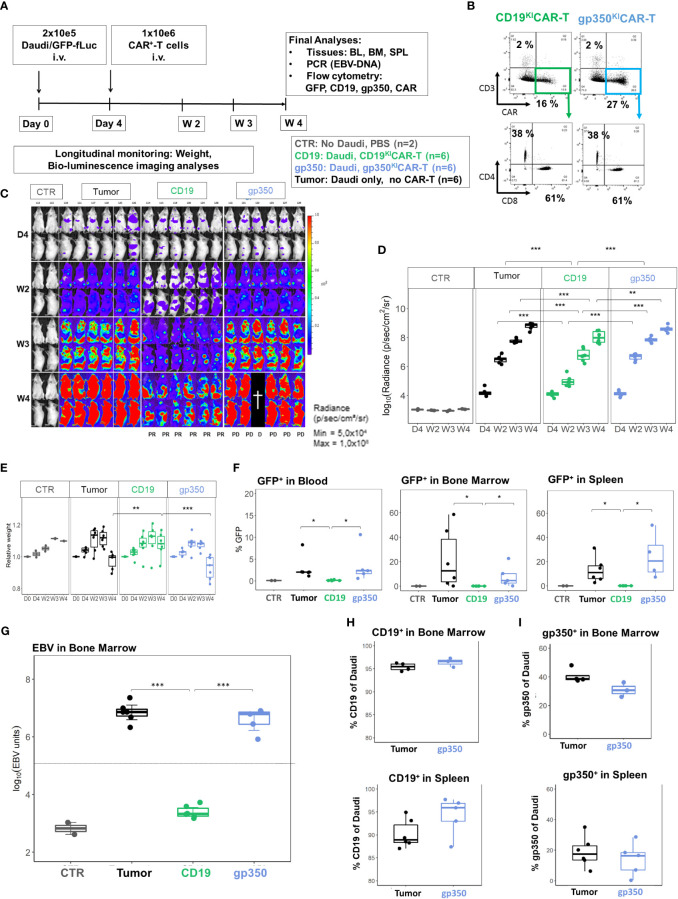
CD19^KI^CAR-T cells showed therapeutic effects against Daudi/fLuc-GFP lymphoma growth, but gp350^KI^CAR-T cells did not. **(A)** Experimental scheme. Female NRG mice (n = 18) were challenged i.v. with 2 × 10^5^ Daudi/fLuc-GFP cells on day 0. Control mice (CTR, n = 2) received PBS. On day 4, the challenged mice were analyzed by bioluminescence imaging (BLI) and then distributed into three cohorts (n = 6 mice each). One cohort was not treated (tumor, depicted in black). One cohort was treated with 1 × 10^6^ CAR^+^ CD19^KI^CAR-T cells (CD19, depicted in green), and the third cohort was treated with 1 × 10^6^ CAR^+^ gp350^KI^CAR-T cells (gp350, depicted in blue). The experiment was terminated at 4 weeks after challenge, and several analyses were performed. **(B)** Flow cytometry analysis of CD19^KI^CAR-T and gp350^KI^CAR-T cells on the day prior to infusion. Upper row: CD3^−^ (y-axis) and CAR-expression (x-axis). Bottom row: cells gated as CD3^−^CAR^+^ were further analyzed for CD4 (y-axis) and CD8 expression (x-axis). **(C)** BLI in frontal and side views for each cohort at day 4, week 2, week 3, and week 4 after challenge. BLI scale is from 5.0 × 10^4^ to 1.0 × 10^8^ p/s/cm^2^/sr. **(D)** Quantified BLI as full body radiance (log scale) shown as boxplots for each cohort and time point. Statistical comparisons were performed *via* ANOVA with Tukey’s *post-hoc* method for each time point (***p < 0.001, **p < 0.01). **(E)** Body weight monitoring. The weights obtained on day 0 for each mouse were used as references for measurements at the subsequent time points. Boxplots for each cohort and time point are shown. Statistical comparisons were performed *via* ANOVA with Tukey’s *post-hoc* method for each time point (***p < 0.001, **p < 0.01). **(F)** Flow cytometry detection of GFP^+^ blasts in blood, bone marrow, and spleen. Boxplots for each cohort analyzed at week 4 are shown in the form of percentages. Statistical comparisons were performed *via* the Kruskal–Wallis test and Tukey’s post-hoc tests (*p < 0.05). **(G)** Detection of EBV DNA in bone marrow. The hatched line indicates the approximate detection limit of EBV units in the samples used for the assay. Boxplots for each cohort analyzed at week 4 are shown on a log scale. Statistical comparisons were performed *via* binomial regression and Tukey’s *post-hoc* tests (***p < 0.001). **(H)** Detection of CD19^+^ cells within GFP^+^/CD20^+^ cells in bone marrow (upper panel) and spleen (lower panel) for non-treated mice or mice treated with gp350^KI^CAR-T cells. Each cohort is shown in the form of boxplots. **(I)** Detection of gp350^+^ cells in bone marrow (upper panel) and spleen (lower panel) for non-treated mice or mice treated with gp350^KI^CAR-T cells. Each cohort is shown in the form of boxplots. Only samples with readily detectable GFP^+^CD20^+^ populations (tumor-only and gp350^KI^CAR) are shown; this resulted in varying sample numbers (SPL: n = 6, tumor-only; n = 5, gp350^KI^CAR; in BM: n = 4, tumor-only; n = 3, gp350^KI^CAR).

In the next step, we established BL xenograft models in order to evaluate the therapeutic effects of ^KI^CAR-T cells *in vivo*. The Daudi/fLuc-GFP model was established first. After pilot experiments to set up the lymphoma challenge, we selected a challenge format consisting of 2 × 10^5^ tumor dose i.v., as the full course of lymphoma development in mice could be followed by BLI from day 4 after challenge for up to 4 weeks (**Figure 5A**). The terminal analyses were then set at 4 weeks after challenge, as at this point the challenged mice started to meet the predefined euthanasia criteria in the approved animal protocols (see Material and Methods section). BLI analyses on day 4 post-challenge showed signals in the anatomical regions of the bones and liver (**Figure 5B**). Two weeks after challenge, the BLI signals were distributed throughout the body of the mice. After 4 weeks, the signals were highly perceptible in several anatomical regions, and particularly in the femurs, hips, and ribs. Female mice showed higher signals than male mice (**Figure 5B**). After euthanasia, BLI was repeated for direct visualization of the abdominal cavity. Macroscopic examination combined with BLI demonstrated that the signals were high in the LNs, kidneys, and ovaries (**Figure 5C**). Tumors could be sporadically visualized in the kidneys (**Figure 5D**). Age-matched non-challenged control mice showed no tumor development. BM smears stained with Giemsa showed the presence of small lymphocytes in control NRG mice and of large blasts in mice challenged with Daudi (**Figure 5E**). Flow cytometry analyses of SPL and BM samples confirmed the detection of GFP^+^ cells. LNs were easily detectable and largely infiltrated with GFP^+^ blasts (**Figure 5F**). After gating on the GFP^+^ cells, flow cytometry analyses further showed the persistent expression of the B-cell markers CD19 and CD20 and, to a lesser extent, of gp350 on the cell surface of the *in vivo* grown lymphoma (**Figures 5G, H**, see gating strategy details in **Figure S4**). Next, the Daudi/fLuc-GFP model was used to evaluate the efficacy of ^KI^CAR-T cells *in vivo* (**Figure 6A**). LS ^KI^CART-T cells were freshly produced, and expression of CAR, CD8, and CD4 was determined during the cell culture process (**Figure 6B**). In parallel, female mice were challenged with 2 × 10^5^ lymphoma cells. After the engraftment was confirmed on day 4, the cohorts were set up based on the BLI signals. Each mouse was infused with 1 × 10^6 KO^TCR^KI^CAR^+^ T cells. Mice were longitudinally monitored by BLI analyses and by weight, and were sacrificed at week 4 after challenge (**Figure 6A**). All challenged mice developed lymphomas, and the cohort treated with CD19^KI^CAR-T cells showed significant delays in BLI signal distribution compared with non-treated mice or mice treated with gp350^KI^CAR-T (**Figures 6C, D**). All the mice treated with CD19^KI^CAR-T cells showed partial responses (PR) (**Figures 6C, D**), and mice maintained constant weight gain overall. In contrast, the non-treated mice and mice treated with gp350^KI^CAR-T cells showed significant weight loss, indicating morbidities (**Figure 6E**). Flow cytometry analyses to detect the presence of GFP^+^ blasts in PBL, BM, and SPL confirmed the therapeutic effects promoted by CD19^KI^CAR-T cells (**Figure 6F**). Analyses of EBV DNA copies in BM showed a significant drop for mice treated with CD19^KI^CAR-T cells (**Figure 6G**). All mice treated with gp350^KI^CAR-T cells showed progressive disease (PD) (**Figures 6C, D**). The persistence of cells expressing CD19^+^ and gp350^+^ within the GFP^+^CD20^+^ blast cell populations recovered from BM and SPL of mice was further assessed by flow cytometry. Interestingly, compared with mice that were only challenged with lymphoma, mice treated with gp350^KI^CAR-T cells showed slightly lower frequencies of cells expressing gp350 (**Figures 6H, I**). Analyses of CD19 and gp350 on lymphoma cells of mice treated with CD19^KI^CAR-T cells were not feasible due to their almost complete elimination. The expression of gp350 was reduced on blasts, which could be associated with mechanisms of antigen escape, reducing the putative therapeutic effects of gp350^KI^CAR-T cells. Flow cytometry analyses of BM and SPL for detection of ^KI^CAR-T cells in mice challenged with Daudi were attempted twice, but ^KI^CAR-T cells were barely detectable on terminal analyses (less than 0.1%, data not shown). Currently, we do not have an explanation for this; this phenomenon could be assessed in the future by cross-sectional analyses.

### Evaluation of ^KI^CAR-T cells in the Jiyoye/fLuc-GFP xenograft model

**Figure 7:**
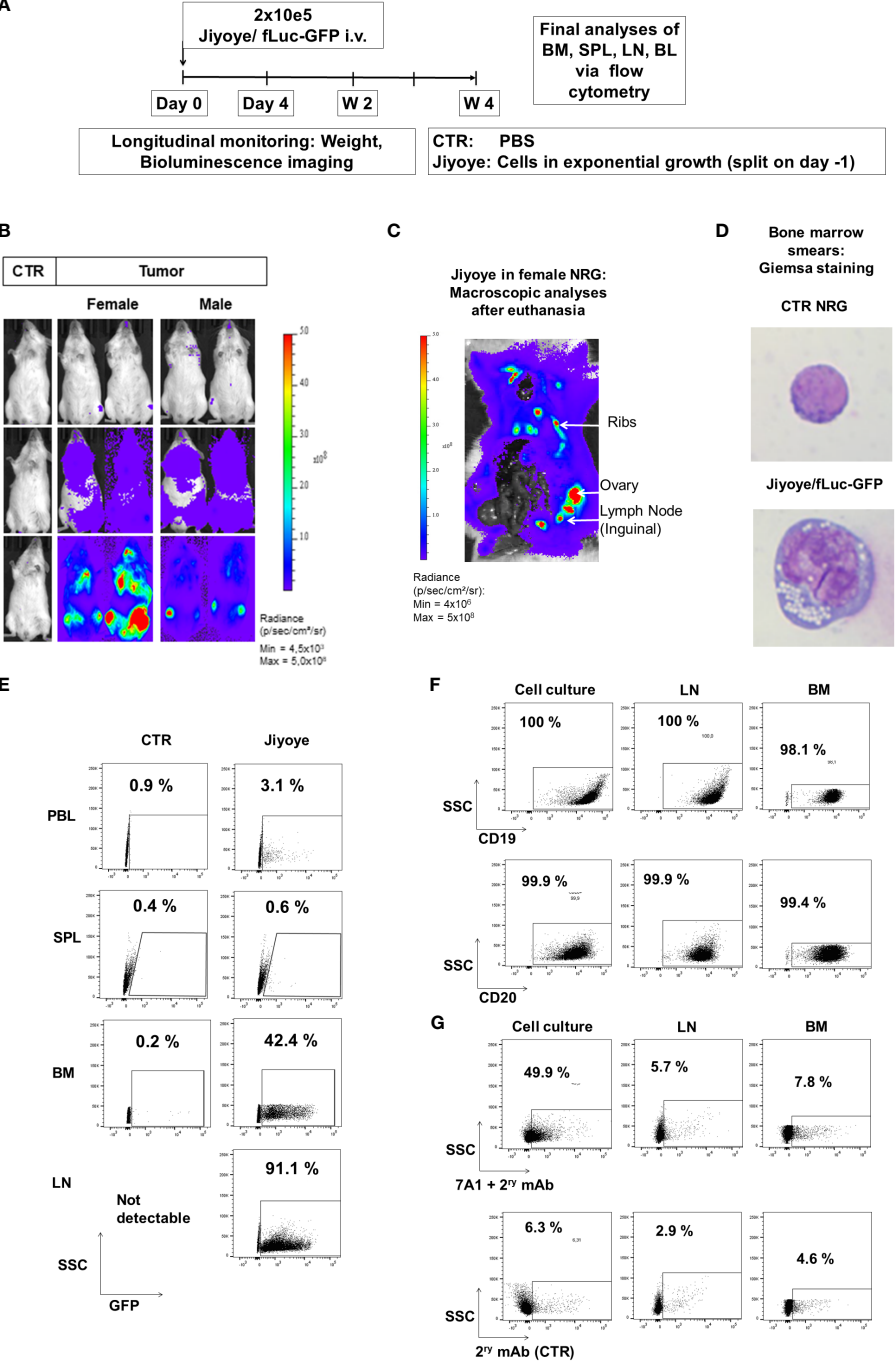
Jiyoye/fLuc-GFP engrafted and disseminated in bones and several organs of NRG mice and maintenance of surface expression of GFP, CD19, CD20, and lower expression of gp350 *in vivo*. **(A)** Experimental scheme. Nod.Rag.Gamma (NRG) mice were challenged with 2 × 10^5^ Jiyoye/fLuc-GFP cells (i.v. injection; females, n = 3; males, n = 3; non-challenged PBS control, CTR, n = 1). **(B)** Representative examples of two challenged female and male mice and a control mouse are shown. Mice were analyzed longitudinally *via* bioluminescence imaging (BLI), and the experiment was then terminated. BLI is shown at 4 days, 2 weeks, and 4 weeks after challenge (frontal view; scale from 4,5 × 10³ to 5 × 10^8^ p/s/cm^2^/sr). **(C)** Post-mortem abdominal BLI of a female mouse developing lymphoma infiltrating the bones, lymph nodes, and ovary (scale: 4 × 10^6^ to 5 × 10^8^ p/s/cm^2^/sr). **(D)** Bone marrow smears stained with Giemsa. Mouse normoblast observed in control mice and cells with typical morphology of lymphoma observed in one mouse challenged with Jiyoye/fLuc-GFP cells. Original magnification ×1,000. **(E)** Flow cytometry analyses of peripheral blood (PBL), spleen (SPL), bone marrow (BM), and lymph nodes (LNs). GFP expression (shown on the x-axis) was maintained in blasts; 10,000 events are shown. **(F)** Flow cytometry analyses of Jiyoye/fLuc-GFP cells maintained in culture or explanted from LNs and BM. GFP^+^ blasts express CD19 and CD20; 10,000 events are shown. **(G)** Flow cytometry analyses of Jiyoye/fLuc-GFP cells maintained in culture or explanted from LNs and BM. Subpopulations of GFP^+^CD20^+^ blasts expressed low frequencies of gp350. The samples were stained with 7A1 primary mAb for gp350 detection and secondary Ab (upper row) or with secondary Ab only (bottom row); 10,000 events are shown per plot. Unstained samples were used as references.

**Figure 8:**
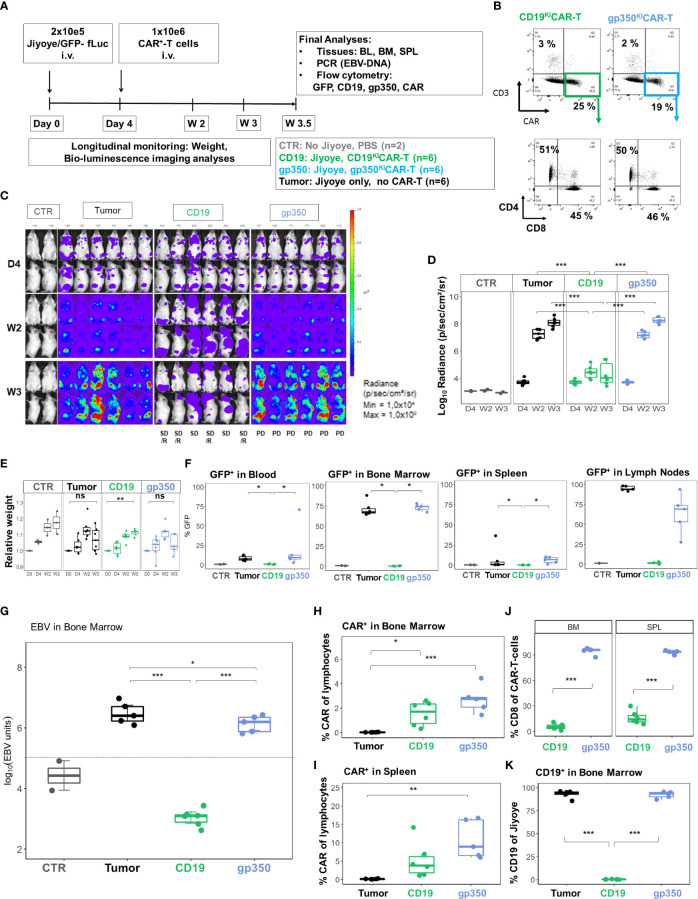
CD19^KI^CAR-T cells showed therapeutic effects against Jiyoye/fLuc-GFP lymphoma growth with accumulation of CD4^+^CAR-T cells, whereas gp350^KI^CAR-T cells reduced EBV DNA load with accumulation of CD8^+^CAR-T cells. Therapeutic administration of ^KI^CAR-T cells into mice challenged with Jiyoye/fLuc-GFP lymphoma. **(A)** Experimental scheme. Female NRG mice (n = 18) were challenged i.v. with 2 × 10^5^ Jiyoye/fLuc-GFP cells on day 0. Control mice (CTR, n = 2) received PBS. On day 4, the challenged mice were analyzed *via* bioluminescence imaging (BLI) and distributed into three cohorts (n = 6 mice each). One cohort was not treated (tumor, depicted in black). One cohort was treated with 1 × 10^6^ CAR^+^ CD19^KI^CAR-T cells (CD19, depicted in green), and the third cohort was treated with 1 × 10^6^ CAR^+^ gp350^KI^CAR-T cells (gp350, depicted in blue). The experiment was terminated at 3.5 weeks after challenge for several analyses because some mice were moribund. **(B)** Flow cytometry analysis of CD19^KI^CAR-T and gp350^KI^CAR-T cells on the day prior to infusion. Upper row: CD3^−^ (y-axis) and CAR expression (x-axis). Bottom row: cells gated as CD3^−^CAR^+^ were further analyzed for CD4 (y-axis) and CD8 expression (x-axis). **(C)** BLI in frontal and side views for each cohort at day 4, week 2, and week 3 after challenge. BLI scale is from 1 × 10^4^ to 1 × 10^9^ p/s/cm^2^/sr. **(D)** Quantified BLI as full body radiance (log scale) shown in the form of boxplots for each cohort and time point. Statistical comparisons were performed *via* ANOVA with Tukey’s *post-hoc* method for each time point (***p < 0.001). **(E)** Body weight monitoring ns indicates not significant. The weight measured on day 0 for each mouse was used as reference for measurements taken at subsequent time points. Boxplots for each cohort and time point are shown. Statistical comparisons were performed *via* ANOVA with Tukey’s *post-hoc* method for each time-point **p < 0.01). **(F)** Flow cytometry detection of GFP^+^ blasts in blood, bone marrow, spleen, and lymph nodes. Boxplots for each cohort analyzed at week 4 are shown in the form of percentages. Statistical comparisons were performed *via* Kruskal–Wallis test and Tukey’s post-hoc tests (***p < 0.001, **p < 0.01, *p < 0.05). **(G)** Detection of EBV DNA in bone marrow. The hatched line indicates the approximate detection limit of EBV units in the samples used for the assay. Boxplots for each cohort analyzed at week 4 are shown on a log scale (tumor: n = 5, one sample not available; CD19: n = 6: gp350: n = 5). Statistical comparisons were performed *via* binomial regression and Tukey’s *post-hoc* tests (***p < 0.001, *p < 0.05). **(H, I)** Detection of CAR^+^ cells in bone marrow **(H)** and spleen **(I)** for non-treated mice or mice treated with CD19^KI^CAR-T cells or with gp350^KI^CAR-T cells. Data were analyzed *via* ANOVA (type 2) and Tukey’s *post-hoc* tests (***p < 0.001, **p < 0.01, *p < 0.05). **(J)** Frequencies of CD8^+^CAR^+^ cells detected in the spleen and bone marrow of mice treated with CD19^KI^CAR-T cells or with gp350^KI^CAR-T cells. Data were analyzed *via* ANOVA (type 2) and Tukey’s *post-hoc* test (***p < 0.001). **(K)** Detection of CD19^+^ cells in bone marrow for non-treated mice or mice treated with CD19^KI^CAR-T cells or with gp350^KI^CAR-T cells. Each cohort is shown in the form of boxplots. Data were analyzed *via* ANOVA (Type II) and Tukey’s *post-hoc* tests (***p < 0.001). Only samples with readily detectable GFP^+^CD20^+^ populations are shown.

We subsequently developed the Jiyoye *in vivo* model to assess the effects of the ^KI^CAR-T cells (**Figure 7A**). BLI analyses after Jiyoye/fLuc-GFP challenge showed lymphoma development, which occurred particularly quickly in female mice (**Figure 7B**). The BLI signal was mostly localized in the bones, LNs, and ovaries (**Figure 7C**). Analyses of BM smears confirmed the presence of cells with typical BL morphology (**Figure 7D**). GFP^+^ blasts were most prevalent in BM and LNs (**Figure 7E**). Lymphoma GFP^+^ blasts expressed CD19 and CD20 (>99%) (**Figure 7F**). Compared to lymphoma cells maintained in culture, only a small fraction of the GFP^+^ cells recovered from LNs or BM showed detectable gp350 expression (**Figure 7G**). The Jiyoye model was then used to test CD19^KI^CAR-T and gp350^KI^CAR-T cells *in vivo* using a similar scheme as previously employed for the Daudi model; 1 × 10^6 KI^CAR-T cells were applied i.v. per mouse (**Figure 8A**). The cells were used while fresh after production (CAR positivity for CD19^KI^CAR-T was 25% and for gp350^KI^CAR-T cells 19%; CD4/CD8 ratios approximately 50/50) (**Figure 8B**). Longitudinal analyses showed a significant reduction in BLI signal for mice treated with CD19^KI^CAR-T cells compared with the other cohorts (**Figures 8C, D**). Comparing the signals between weeks 2 and 3, for most mice, the lymphoma spread was reduced (**Figures 8C, D**). Challenged and non-treated mice or mice treated with gp350^KI^CAR-T cells showed major weight loss on week 3 after challenge (**Figure 8E**). At week 3.5, one mouse of the gp350^KI^CAR-T cohort died, and at this point, the experiment was terminated to reduce the suffering of the mice and to allow harvesting of the tissues. The frequencies of GFP^+^ blasts in PBL, BM, SPL, and LNs were significantly lower for mice treated with CD19^KI^CAR-T cells compared with the other cohorts (**Figure 8F**). Detection of EBV DNA in BM was significantly lower for mice treated with CD19^KI^CAR-T cells (***p < 0.001, n = 6) and for the surviving mice treated with gp350^KI^CAR-T cells (*p < 0.05, n = 5) compared with challenged non-treated mice (n = 6) (**Figure 8G**). Higher frequencies of gp350^KI^CAR-T cells T cells than CD19^KI^CAR-T cells were observed in BM and SP (**Figures 8H, I**). While gp350^KI^CAR-T cells were >90% CD8^+^CAR^+^, the frequencies of CTLs within CD19^KI^CAR-T were significantly lower, and <10% were CD8^+^CAR^+^ (**Figures 8J**). Remarkably, GFP^+^CD20^+^ Jiyoye blasts recovered from BM of mice treated with CD19^KI^CAR-T cells showed loss of CD19 expression (**Figure 8K**). Therefore, the Jiyoye BL model confirmed the therapeutic activity of CD19^KI^CAR-T cells. Further, the stable disease could be explained by the CD19 antigen loss. However, persisting with gp350^KI^CAR-T cell therapy was associated with a reduction of the EBV DNA load, although this did not produce therapeutic effects, and the persisting ^KI^CAR-T cells were almost exclusively CD8^+^ cells.

### Analyses of ^KI^CAR-T cells for expression of activation/exhaustion markers

**Figure 9:**
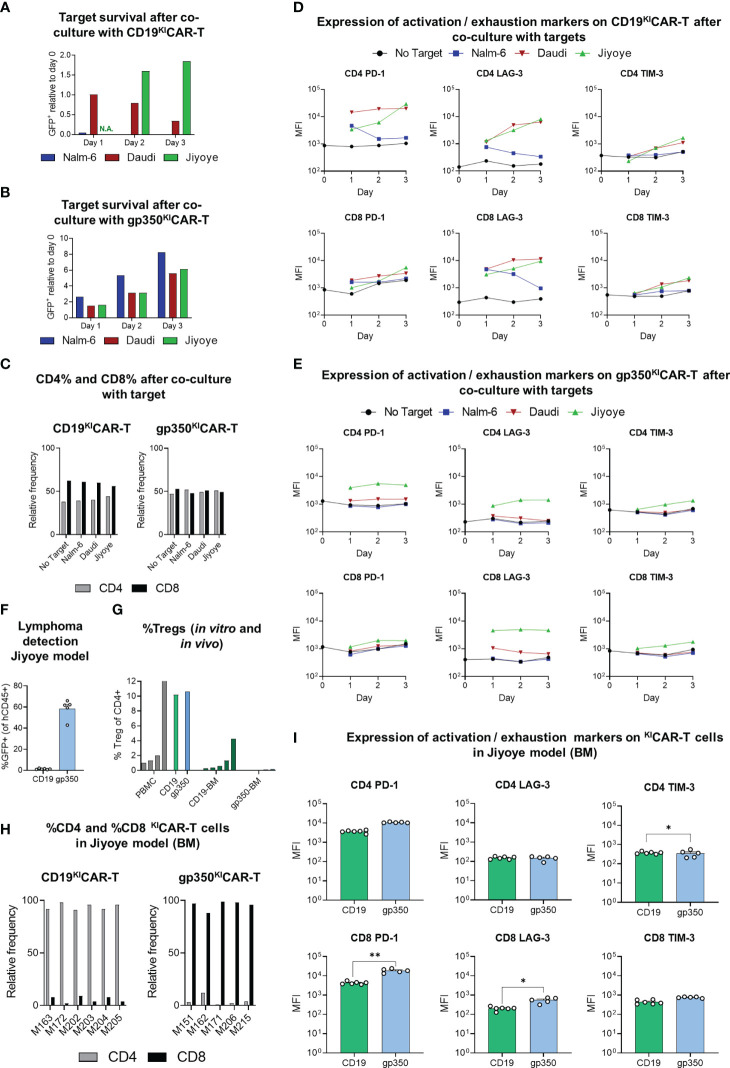
Expression of activation/exhaustion markers and T_reg_ frequencies showing differences for CD19^KI^CAR-T and gp350^KI^CAR-T cells. ^KI^CAR-T cells that were exposed to lymphoma cell lines *in vitro* **(A–E)** or *in vivo* **(F–I)** were compared. **(A, B)** Survival of GFP^+^ targets (CD19^+^gp350^−^ Nalm-6, CD19^+^gp350^+^ Daudi, or CD19^+^gp350^+^ Jiyoye) after co-culture with **(A)** CD19^KI^CAR-T cells or with **(B)** gp350^KI^CAR-T cells at 1:1 E:T ratio for 1, 2, or 3 days. The cells were harvested at different time points and analyzed *via* flow cytometry for quantification of the frequencies of GFP^+^ targets. Day 0 was used as a reference to show the relative decrease or increase in frequencies of the targets in the cultures. **(C)** Relative frequencies of CD4^+^ or CD8^+^ effectors (left panel: CD19^KI^CAR-T cells; right panel: gp350^KI^CAR-T) cultured for 3 days with no target or with addition of the target cell lines. **(D, E)** Longitudinal analyses of expression of activation/exhaustion markers (left: PD-1; middle: LAG-3; right: TIM-3) on CD4^+^ (top) and CD8^+^ (bottom) for **(D)** CD19^KI^CAR-T cells or **(E)** gp350^KI^CAR-T cells (mean fluorescence intensity, MFI log_10_). Expression was measured before culture (day 0) and repeatedly every 24 h (days 1–3) for CAR-T cells co-cultured with different targets (E:T ratio 1:1). ^KI^CAR-T cells were gated as GFP-negative and CAR-positive cells (see **Figure S6**). **(F–I)** Re-analyses *via* flow cytometry of cryopreserved bone marrow specimens of mice transplanted with Jiyoye. **(F)** Infiltration of Jiyoye, quantified as percentage of GFP^+^ cells within all identified human CD45^+^ cells (CD19 ^KI^CAR-T, n = 6; gp350 ^KI^CAR-T, n = 5; mean + SEM). **(G)** Frequencies of regulatory T cells (T_reg_) for healthy donor PBMCs (gray, n = 5), for *ex vivo* expanded ^KI^CAR-T cells (CD19: green; gp350: blue; n = 1 each), and CAR-T cells detected in bone marrow samples of mice challenged with Jiyoye/fLuc-GFP (CD19: dark green, n = 6; gp350: dark blue, n = 5). T_reg_ cells were identified as CD4^+^ FoxP3^+^ (gating strategy: **Figure S7**) and are depicted in the form mean + SEM where applicable. **(H)** Relative frequencies of CD4^+^ and CD8^+^ subpopulations of CAR^+^ T cells detectable in bone marrow samples (left: CD19^KI^CAR-T cells, n = 6; right: gp350^KI^CAR-T cells, n = 5). **(I)** Flow cytometry analysis of bone marrow samples of mice challenged with Jiyoye/fLuc-GFP. Expression of various exhaustion markers (left: PD-1; middle: LAG-3; right: TIM-3) for CD4 (top) and CD8 (bottom) ^KI^CAR-T cells (CD19: green, n = 6; gp350: blue, n = 5; mean + SEM). CAR-T cells were gated as GFP-negative and CAR-positive cells (gating strategy: **Figure S6**). Significance was assessed *via* Student’s t-test (**p < 0.01, *p < 0.05).

We have previously shown in a humanized mouse model recapitulating EBV^+^ PTLD that two immunologic hallmarks associated with tumor progression are the increased expression of activation/exhaustion markers on CD8^+^ T cells (PD-1, LAG-3, and TIM-3) and accumulation of CD4^+^ T_regs_ (27) (28). Therefore, we first assessed *in vitro* whether co-culture of CD19^KI^CAR-T cells or gp350^KI^CAR-T cells with the EBV^+^ BL lines at 1:1 E:T ratio would result in upregulation of the activation/exhaustion makers. EBV-negative Nalm-6 acute lymphoblastic leukemia cells expressing CD19 were included as controls. The co-cultures were maintained for 3 days and were analyzed daily for target cell killing, CD4/CD8 ratios, and expression of the exhaustion markers. CD19^KI^CAR-T cells eliminated the Nalm-6/fLuc-GFP cells, progressively killed the Daudi/fLuc-GFP cells, and were not effective at killing the Jiyoye/fLuc-GFP cells (**Figure 9A**). Under these experimental conditions, gp350^KI^CAR-T cells did not react against Nalm-6/fLuc-GFP cells, and only reduced the growth of Daudi/fLuc-GFP and Jiyoye/fLuc-GFP cells (**Figure 9B**). At the end of the cultures, CD19^KI^CAR-T cells showed higher frequencies of CD8^+^ than CD4^+^ T cells, but this trend was not observed for gp350^KI^CAR-T cells (**Figure 9C**). CD19^KI^CAR-T cells exposed to Nalm-6 cells showed low expression of the exhaustion markers, similar to the baseline control (without target cells) (**Figure 9D**). CD19^KI^CAR-T cells incubated with either Daudi or Jiyoye showed similar upregulation of PD-1 on CD4^+^ cells and of PD-1 and LAG-3 on CD8^+^ cells (**Figure 9D**, gating strategy details in **Figure S6**). In contrast, gp350^KI^CAR-T cells exposed to Nalm-6 or to Daudi cells did not show upregulation of exhaustion markers. However, gp350^KI^CAR-T cells co-cultured with Jiyoye, similarly to the outcome with CD19^KI^CAR-T cells, showed upregulation of PD-1 and LAG-3 on the CD4^+^ and of LAG-3 on CD8^+^ T cells (**Figure 9E**). Thus, overall, the *in vitro* data indicated that Jiyoye promoted the exhaustion of both CD19^KI^CAR-T and gp350^KI^CAR-T cells. Subsequently, we evaluated the effects of Jiyoye *in vivo*. We used the cryopreserved BM samples shown in **Figure 8F** of mice challenged with Jiyoye and treated with ^KI^CAR-T cells. GFP^+^ blasts were almost undetectable in mice treated with CD19^KI^CAR-T cells, but were present in mice treated with gp350^KI^CAR-T cells (**Figure 9F**). On the other hand, compared with CD8^+^CD19^KI^CAR-T cells, significantly higher expression of PD-1 and LAG-3 was observed in CD8^+^gp350^KI^CAR-T. Thus, from this series of analyses, we concluded that the short 3-day co-culture of Jiyoye with ^KI^CAR-T cells promoted their activation and exhaustion in a similar way. However, their longer exposure to Jiyoye *in vivo* resulted in a dichotomy between higher occurrences of CD4^+^CD19^KI^CAR-T_regs_ and exhaustion of CD8^+^gp350^KI^CAR-T cells (see **Table S2**).

### Analyses of ^KI^CAR-T cells expanded in mice challenged with Jiyoye for detection of T_regs_

We used BM samples recovered from mice challenged with Jiyoye to detect T_regs_ by flow cytometry, defined as CD4^+^FoxP3^+^ (**Figure 9G**, see gating strategy details in **Figure S7**). As staining controls, we used PBMCs (positive controls, range of 1.01%–12.70% T_regs_) or ^KI^CAR-T cells maintained in culture (approximately 10% T_regs_). Mice challenged with Jiyoye and treated with CD19^KI^CAR-T cells showed significantly higher T_reg_ frequencies (range of 0.05%–4.25% T_regs_) than mice treated with gp350^KI^CAR-T cells (less than 0.15% T_regs_). This was correlated with the previously seen bias toward accumulation of CD4^+^CD19^KI^CAR-T cells and CD8^+^CD19^KI^CAR-T cells (**Figure 9H**, see **Table S2**). The significantly higher rate of detection of TIM-3^+^CD4^+^ cells within CD19^KI^CAR-T than gp350^KI^CAR-T cells represents another interesting finding since TIM-3 expression is a marker expressed on functional T_regs_ in human tumors (29).

## Discussion

EBV is highly prevalent worldwide in adults, exhibiting life-long persistence, and the infection is associated with several types of debilitating diseases, from acute infection to autoimmune conditions and ultimately cancer. Thus far, no form of cell therapy or immunotherapy has been approved to treat EBV-related malignancies (30). Here, we explored edited ^KI^CAR-T cells as a future therapy against EBV^+^ BL. Overall, our results indicated the feasibility of CD19^KI^CAR-T cell therapy, and we also observed intriguing outcomes regarding ^KI^CAR-T cell dynamics *in vivo*.

Historically, the generation of CAR-T cells has largely relied on retroviral and lentiviral vectors (14). We generated CD19^KI^CAR-T cells and gp350^KI^CAR-T cells using non-viral CRISPR products (Cas enzyme, gRNAs, and DNA HDRTs) in combination with an up-scalable MaxCyte ExPERT ATx^®^ electroporation technology. Using an LS production protocol, we achieved high rates of TCR-KO (98%) and detectable CAR-KI (20%), and the T cells were viable and expanded. Although viral vectors used for CAR-T cell productions currently result in higher CAR expression (50%–80%) (15) (31), several ongoing improvements in gene editing KI are promising, such as the use of less toxic ssDNA donor templates (32). Shy et al. recently demonstrated that hybrid ssDNA donors containing double-stranded end modifications achieve >40% CAR KI rates at the clinical scale with the MaxCyte ExPERT GTx^®^ device (33). In addition, the design of the HDRTs and inclusion of tags can be optimized to reduce their size and facilitate the enrichment of ^KI^CAR cells in order to improve the purity of the product. Further, PGA and other anionic adjuvants can reduce toxicity and increase the efficacy of KI with large dsDNA donor templates (34). Although the field is still assimilating current technical improvements, ^KI^CAR-T production using methods compliant with good manufacturing practice (GMP), employing either the ExPERT GTx^®^ Flow Electroporation^®^ system or fully automated and closed manufacturing systems (35–37), will in due course be established for clinical products. In terms of the quality control (QC) analyses of the ^KI^CAR-T cells, we have not assessed off-target editing or chromosomal aberrations such as large deletions or translocations. Nevertheless, identification of potential off-target sites of the *TRAC* gRNA and CD19CAR HDRT used in this study was previously performed *in silico*, *in cellulo* (20), and *via* whole genome sequencing (38), and no off-target editing was detectable in primary human T cells. Therefore, the risks of insertional mutagenesis of HDR-based gene editing are likely lower than those of viral vectors (32), the production costs are likely to be lower, and the clinical use of ^KI^CAR-T cells will be expanded to supply the clinical demand.

The studies presented here aim specifically to demonstrate the feasibility and potency of ^KI^CAR-T cells against EBV^+^ BL. This was initially demonstrated *in vitro* after co-culture of the two modalities of ^KI^CAR-T cells with Daudi (type I EBV) and Jiyoye (type 2 EBV) BL lines followed by analyses of cytotoxicity and IFN-γ secretion. Our results showed consistently higher *in vitro* potency of CD19^KI^CAR-T cells than gp350^KI^CAR-T. The differences between the CAR constructs were negligible, and the expression of the CARs was similar. Since CD19 and gp350 are very dissimilar targets with different biological functions (cellular versus viral), we cannot conclude that these results rely solely on the scFv binding affinity to their respective targets. Thus, the most plausible explanation is the high and homogeneous expression of CD19, compared with the weaker and variable expression of gp350 on the BL lines used for the studies (**Figure 3B**).

Next, we developed experimental systems to compare the potency of the ^KI^CAR-T cells against BL. Cell line-derived xenograft (CDX) tumor models are convenient as initial models. This is particularly true if the cell lines are traceable with characteristics associated with the tumor entity and can be obtained commercially from repositories for comparative studies in separate laboratories. CD19CAR-T cells have been commonly tested *in vivo* in NOD-*scid-IL2rγ^(−/−)^* (NSG) and in NOD/Shi-*scid IL2rγ^(−/−)^* (NOG) mice implanted i.v. with ALL Nalm-6 cells expressing fLuc and monitored by optical imaging analyses (39, 40). ALL is well recapitulated with the Nalm-6 model, with the involvement of the bone marrow and also the central nervous system (39). CD19CAR-T cells have also been evaluated in the Raji/fLuc EBV^+^ BL i.v. implantation model in NSG mice (41) (42). However, gp350 expression in Raji is variable and low (less than 5%, data not shown). For our studies, we used the NRG mouse strain because these mice show the development of lymph node-like structures infiltrated with mature human T and B cells in fully humanized mice (43, 44). NRG female mice engrafted with Daudi or Jiyoye cells showed faster tumor development than male mice, leading to morbidity at 3–4 weeks after challenge. The Daudi/fLuc-GFP cells consistently engrafted in 100% of the mice, and on day 4 after challenge, lymphoma spread could be monitored in lymphatic tissues and several organs, particularly the bones, kidneys, and ovaries. The Jiyoye/fLuc-GFP cells also resulted in 100% engraftment, but the lymphoma spread was initially in bones and later in lymph nodes, whereas the spread of lymphoma in the spleen was negligible. Despite these divergent bio-distribution patterns, expression of GFP, CD20, CD19, and gp350 was detectable in lymphoma cells, and EBV DNA was detectable in bone marrow samples.

All mice treated with CD19^KI^CAR-T cells, for both the Daudi and Jiyoye models, showed responses against BL development (measured by BLI, flow cytometry, and detection of EBV DNA). The overall responses against Jiyoye were more profound than those against Daudi lymphoma. In fact, therapeutic responses were associated with reduced expression of CD19 on the blasts, indicating antigen escape. None of the mice succumbed within the period of the experiments; nonetheless, residual lymphoma could be detected by BLI and flow cytometry for both models, indicating residual disease.

None of the mice challenged with Daudi showed human T cells or ^KI^CAR-T cells above background levels in the BM or SPL. This could have resulted from exhaustion *via* PD-1 upregulation, as indicated by the co-culture experiments of Daudi cells with ^KI^CAR-T cells. In contrast, CD19^KI^CAR-T cells were detectable in mice challenged with Jiyoye, and these were mostly CD4^+^. Remarkably, within the CD4^+^ population, some cells showed immune phenotypic characteristics of T_regs_ (**Figure 9G**). We do not have a mechanistic explanation for this effect, but we have observed an accumulation of CD4^+^ T_regs_ and CD8^+^ T cells with exhaustion patterns in fully humanized mouse models of EBV^+^ PTLD (27, 28). This bias toward CD4 positivity in CAR-T cell dynamics should be kept in mind, as Mackall and Maus’s groups using CD19CAR-T cells to treat leukemia and lymphoma have detected the presence of CD4^+^ T_regs_ within the CAR-T cells products generated with viral vectors, and higher frequencies of T_regs_ are associated with poorer outcomes (45, 46). This also relates to early clinical studies with tandem CD19/22CAR-T cells used to treat refractory BL, with objective responses in 3/6 treated patients. The patients with bulky disease and shorter survival times had higher levels of T_regs_ (47). Therefore, it is tempting to conjecture that, although CD19CAR-T cells are overall effective at debulking the tumor, the residual tumor environment may trigger the outgrowth of CAR-T cells with T_reg_ immunosuppressive properties.

Despite the encouraging *in vitro* potency results, treatment of mice with gp350^KI^CAR-T cells did not show therapeutic responses against lymphoma development *in vivo*. As mentioned above, the main reason for the lower efficacy of gp350^KI^CAR-T cells compared with CD19^KI^CAR-T cells was most likely the lower and variable gp350 expression on lymphoma cells. BL cells showed lower expression than CD19 *in vitro* and only a subset of cells expressed gp350 (**Figure 3B**). Further, gp350 expression was reduced *in vivo*, indicating antigen loss (**Figures 5H**, **7G**), and, after administration of gp350^KI^CAR-T in mice, gp350 expression was further reduced, indicating antigen escape (**Figure 6I**). Conversely, CD19 expression was higher and uniform on the BL cells *in vitro* and *in vivo*, although we were also able to observe antigen escape after CD19^KI^CAR-T cell administration (**Figure 8K**).

The decreased EBV DNA load observed after gp350^KI^CAR-T cell administration is intriguing and supports the likelihood of gp350 antigen escape, possibly mediated by a switch of the cells from EBV lytic toward latent status. As the cells with the highest numbers of EBV copies were killed, the lymphoma with reduced EBV load (or showing lower lytic patterns) survived. EBV lytic induction therapy is an emerging virus-targeted therapeutic approach that exploits the presence of EBV in tumor cells to confer specific killing effects against EBV-associated malignancies. Different classes of chemical compounds, so-called EBV lytic inducers, are in preclinical or clinical development against EBV^+^ nasopharyngeal carcinoma (NPC) (48, 49). An interesting approach would be the combination of lytic inducers and gp350^KI^CAR-T cell therapies; on the one hand, this would stimulate EBV reactivation with gp350 upregulation, while gp350^KI^CAR-T cells would more readily detect them. In fact, we recently showed that gp350 can be detected in NPC and that gp350CAR-T cells generated with lentiviral vectors in compliance with GMP reduce NPC tumor load in a preclinical mouse model used to evaluate the pharmacodynamics and pharmacokinetics of the first-in-class cell product reaching clinical trials (Zhang et al., in press in *Frontiers in Immunology* https://doi.org/10.3389/fimmu.2023.1103695). An additional potential strategy to augment the functionality of gp350^KI^CAR-T cells against BL would be to include additional targets, such as a tandem CD19gp350^KI^CAR-T cell, but this was out of the scope of the current work.

Another fascinating finding noticed in the Jiyoye model was the conspicuously high frequencies of CD8^+^ gp350^KI^CAR-T cells compared to CD4^+ KI^CAR-T cells. In previous work, we have demonstrated a systemic exhaustion phenotype of CD8^+^ T cells, resulting in upregulation of the inhibitory checkpoints PD-1, TIM-3, and LAG-3 in a fully humanized mouse model of EBV^+^ PTLD (27). Nonetheless, in this model, while the CD4^+^ T cells vanished, the CD8^+^ T cells expanded *in vivo* and could still recognize EBV antigens *in vitro*. Administration of immune checkpoint inhibitor targeting PD-1 (pembrolizumab) in humanized mice with EBV^+^ PTLD exacerbated T_reg_ accumulation and tumor development (28). Of course, ^KI^CAR-T cells could be further edited to knock out expression of PD-1, LAG-3, or TIM-3, but in view of the putative EBV interferences, this will require careful evaluation.

The field of non-viral gene-edited CAR-T cells is currently in its infancy, and we are still learning about how best to achieve precise insertions of CARs into defined genomic loci and handle combinatorial gene knock-outs and knock-ins (14). Although the rate of successful CRISPR/Cas insertion of the CAR shown here (in the range of 20%) could be considered low using virally transduced CAR-T cells as a point of comparison, we must remember that in the first successful clinical trial of CD19CAR-T cells, reported by Carl June and collaborators, lentiviral transduction efficacy was in the range of just 5%–20% (transduction efficiency defined as scFv expression) (50). As we know, although the LV transduction efficacy has improved in the meantime to approximately 50%–80%, the manufacturing of LVs remains complex and costly and is a major limitation for clinical-grade CAR-T cell production. We expect that gene editing reagents and methods will be soon scaled up with high accuracy and under GMP with broad perspectives for T cell engineering to improve their therapeutic efficacy. Incidentally, ^KI^CAR-T cells can also be further edited in the future to knock out the expression of human leukocyte antigens (HLAs) *via* multiplex editing methods for the generation of less immunogenic ^KO^HLA^KO^TCR^KI^CAR-T cells (51). Although additional antigenic proteins would still be present and would not fully preclude the immune responses of the host against an allogenic CD19^KI^CAR-T cell product, this technology can be explored as off-the-shelf gene-edited CAR-T cells for relapsed EBV^+^ BL patients who urgently need them. Further, cryopreserved and banked off-the-shelf CAR-T cells generated with T cells of a single healthy donor can be used to treat several patients with EBV^+^ BL. Indeed, producing many different cryopreserved ^KO^HLA^KO^TCR^KI^CAR-T cell lots from a single healthy donor apheresis product could reduce the costs of CAR-T cell treatments so that they can be made available in equatorial African countries with endemic BL.

In summary, our data obtained in two *in vivo* models consist of compelling results for the further development of CD19^KI^CAR-T cells produced without the need for viral vectors as future therapy against refractory or relapsed EBV^+^ BL. In the meantime, CAR-T cells generated with lentiviral vectors and reactive against EBV-associated NPC will be tested clinically (52). Our findings also call attention to possible deviations of CAR-T cell dynamics toward T_regs_, which requires future mechanistic evaluation in experimental models and careful clinical examination. Although gp350^KI^CAR-T cells did not show therapeutic effects *in vivo* against EBV^+^ BL, they remain to be further examined in experimental models of EBV^+^ PTLD and NPC (52), and eventually combined with compounds inducing EBV reactivation and higher gp350 expression. Ultimately, gene editing strategies can be designed to optimize several aspects of CAR-T cell therapies, such as potency, specificity, and persistency, and for large-scale production of off-the-shelf cells for distribution to clinical centers worldwide.

## Data availability statement

The raw data supporting the conclusions of this article will be made available by the authors, without undue reservation.

## Ethics statement

The studies involving human participants were reviewed and approved by Hannover Medical School Ethics Review Board (approval No. 4837). The patients/participants provided their written informed consent to participate in this study. The animal study was reviewed and approved by Niedersächsiches Landesamt für Verbraucherschutz und Lebensmittelsicherheit, Dezernat 33/Tierschutz, LAVES; Protocol No. 33.12-42502-04-21/3791 and 33.12-42502-04-16/2347.

## Author contributions

TB and AP generated the gene-edited CAR-T cells, performed the key *in vitro* and *in vivo* experiments, created the figures and tables, and wrote the drafts of the legends, Material and Methods section, and Results section. MD, AvS, and P-HN performed the co-culture experiments with CAR-T cells and targets and analyzed mouse samples to determine the presence of exhausted T cells and regulatory T cells. DW and JK provided the HDRT for CD19^KI^CAR generation and key technical advice. AR-M provided key technical advice for use of MaxCyte^®^ equipment. LR and AS provided technical assistance. SA provided the PCR analyses of EBV units in BM. MH provided the microscopic analyses of BM smears. CF provided the analyses of cytokines. SRT provided advanced statistical analyses. AB provided consultation for the animal protocols and implementation of animal experiments. MB provided an interpretation of the BL models clinical input regarding BL. RS obtained the funding, supervised the work and collaborations, developed the initial concept of the manuscript, organized and distributed the headings, wrote the Introduction and Discussion sections, and revised the final manuscript. All authors contributed to the article and approved the submitted version.
