# Supplementary material for: Non-viral TRAC-knocked-in CD19KICAR-T and gp350KICAR-T cells tested against Burkitt lymphomas with type 1 or 2 EBV infection: In vivo cellular dynamics and potency
Source: Front Immunol. 2023 Mar 24;14:1086433. doi: 10.3389/fimmu.2023.1086433 (PMC10081580; doi:10.3389/fimmu.2023.1086433)

Supplemental Methods

Giemsa Staining

Slides were airdried and the material was then fixated by submerging in 99.7% methanol and then in May-Grünwald Eosin-Methyleneblue solution (Sigma-Aldrich). The slides were then washed in distilled water, submerged in Giemsa solution (Sigma-Aldrich) and allowed to dry.

Statistical analyses with references

Data were analyzed with the R software (v.4.0.3, R Foundation for Statistical Computing, Vienna, Austria) [1] and GraphPad Prism V7.0 or V9.3 (GraphPad Software, La Jolla, CA, USA). In addition, subgroup data were tested against the hypothesis of normal distribution using the Shapiro-Wilk test and Quantile-Quantile Plots. Data obtained from *in-vitro*-killing assays were analyzed with two-sample *t*-tests, i.e., using sample vs. control or group vs. group comparisons. Exhaustion marker expression comparison between bone marrow samples treated with CD19 and gp350^{KI}CAR-T cells was also analyzed using t-test. Depending on the distribution structure of the *in vivo* data, an ANOVA (normally distributed) or a Kruskal-Wallis test was used as a statistical method. Group contrasts were calculated as post hoc tests and adjusted with the Bonferroni criterion for multiple comparisons. Count data were analyzed with negative binomial regression from the MASS [2] package and reported as log count coefficients or rate ratios with 95% confidence intervals. Post hoc tests were carried out as pairwise comparisons using a Tukey-Kramer test's estimated marginal means of the log count estimates.[3] Results were visualized using the ggplot2 [4] package or GraphPad Prism. The level of significance was set to $p \leq 0.05$.

[1] R Core Team (2020). R: A language and environment for statistical computing. R Foundation for Statistical Computing, Vienna, Austria.

[2] Venables, W. N. & Ripley, B. D. (2002) Modern Applied Statistics with S. Fourth Edition. Springer, New York. ISBN 0-387-95457-0

[3] Russell V. Lenth (2021). emmeans: Estimated Marginal Means, aka Least-Squares Means. R package version 1.5.4.

[4] H. Wickham. ggplot2: Elegant Graphics for Data Analysis. Springer-Verlag New York, 2016.

Supplemental Table 1: Reagents used for flow cytometry analyses.

A Staining of CAR-T cells

Blocking					
Type		Dilution	Cat. Nr.		
IgG (mouse)		10 µg/µl in PBS	I5381-1MG		
Staining					
**Staining or Antigen**	**Dye**	**Clone**	**Dilution**	**Cat. number**	**Source**
Anti-gp350 (rat)	prim.-AB	7A1	1:100	N.A.	Prof. R. Zeidler Helmhotz Munich
AffiniPure Anti-Rat IgG	Alexa Fluor 647	Polyclonal	1:400	212-605-082	Jackson Immuno Research Laboratories
Hu IgG1 for CAR detection	Alexa Fluor 647	Polyclonal	1:100	109-606-170	Jackson Immuno Research Laboratories
Hu CD19	Alexa Fluor 700	HIB19	1:100	302225	Biolegend
Hu CD20	PE	2H7	1:100	302306	Biolegend
Hu CD3	Alexa Fluor700	HIT3a	1:100	300324	Biolegend
Hu CD4	PerCP	OKT4	1:400	317432	Biolegend
Hu CD45	Pacific blue	HI30	1:400	304022	Biolegend
Hu CD8a	PE-Cy7	HIT8a	1:200	300914	Biolegend
Viability Dye eFluor 450	-	-	1:200	65-0863-14	Invitrogen
Viability staining 7AAD	-	-	1:100	130-111-568	Miltenyi Biotec

B Staining of activation/ exhausion markers on CAR-T cells

Antigen	Dye	Dilution	Clone	Cat. number	Source
**Hu IgG1**	AF647	1:50	polyclonal	109-605-098	Jackson ImmunoResearch
**Hu CD4**	PE	1:25	13B8.2	A07751	Beckman Coulter
**Hu CD8**	BV510	1:100	SK1	344732	BIolegend
**Hu PD-1**	BV421	1:25	EH12.2H7	329920	BIolegend
**Hu Tim-3**	APC-Cy7	1:25	F38-2E2	345026	Biolegend
**Hu LAG-3**	PerCp-eFluor710	1:25	3DS223H	46-2239-42	eBioscience

C Staining of T_{regs}

Antigen	Dye	Dilution	Clone	Cat. number	Source
**Hu CD4**	PE-Vio770	1:200	REA623	130-113-227	Miltenyi
**Hu FoxP3**	PE	1:200	206D	320108	Biolegend

Supplemental Table 2: Overview of the CAR-T cell dynamics *in vitro* and *in vivo*.

Assay	$CD19^{KI}$CAR-T	$gp350^{KI}$CAR-T
Large-scale expansion	CD4/CD8 approx 50/50, depends on batch and donor	CD4/CD8 approx 50/50, depends on batch and donor
Co-culture with Daudi	Majority CD8	Majority CD8
Exhaustion Daudi *in vitro*	CD4 LAG3>PD-1>TIM3<br>CD8 LAG3>PD-1>TIM3	No upregulation
Co-culture Jiyoye	Majority CD8	Majority CD8 or 50/50
Exhaustion Jiyoye *in vitro*	CD4 LAG3>PD-1>TIM3<br>CD8 LAG3>PD-1>TIM3	CD4 LAG3>PD-1>TIM3<br>CD8 LAG3>PD-1>TIM3
In vivo Daudi model	CAR-T undetectable	CAR-T undetectable
Exhaustion with Daudi *in vivo*	CAR-T undetectable	CAR-T undetectable
In vivo Jiyoye *in vivo*	Majority CD4	Majority CD8
Exhaustion Jiyoye *in vivo*	CD4 TIM3	CD8 PD-1>LAG3
T_{regs} Jiyoye *in vivo*	Moderate to high.	Not detectable, baseline.

Figure Supplemental 1: (A) Structural differences in the HDRT designs for CD19CAR-BE and gp350CAR-BE versus gp350CAR-HA (related to Figure 1). The gp350CAR-HA contains Gly-Ser-Gly linker in frame with the P2A element, a different membrane export signal and an additional Gly-Asp-Pro-Ala linker in frame with the IgG4 hinge. **(B)** FACS analyses of T cells after electroporation for gene editing showing gating strategy for CAR-T cells. Lymphocyte population gated using FSC and SSC. Singlets were gated using FSC-H and FSC-A. Living cells were gated by staining with fixable viability dye 450. (C) Flow cytometry analyses performed on Day 4 to evaluate the TCR knock-out, which resulted into loss of CD3 detection on the cell surface. Mock cells are $CD3^{+}CAR^{-}$, KO cells only electroporated with gRNA but without HDRT in the RNP complex are $CD3^{-}CAR^{-}$, ^{KI}CAR-T cells show a $CD3^{-}CAR^{+}$ population. Note that the frequency of T cells expressing the CAR and the signal intensity were slightly higher for the gp350CAR-HA than gp350CAR-BE construct.

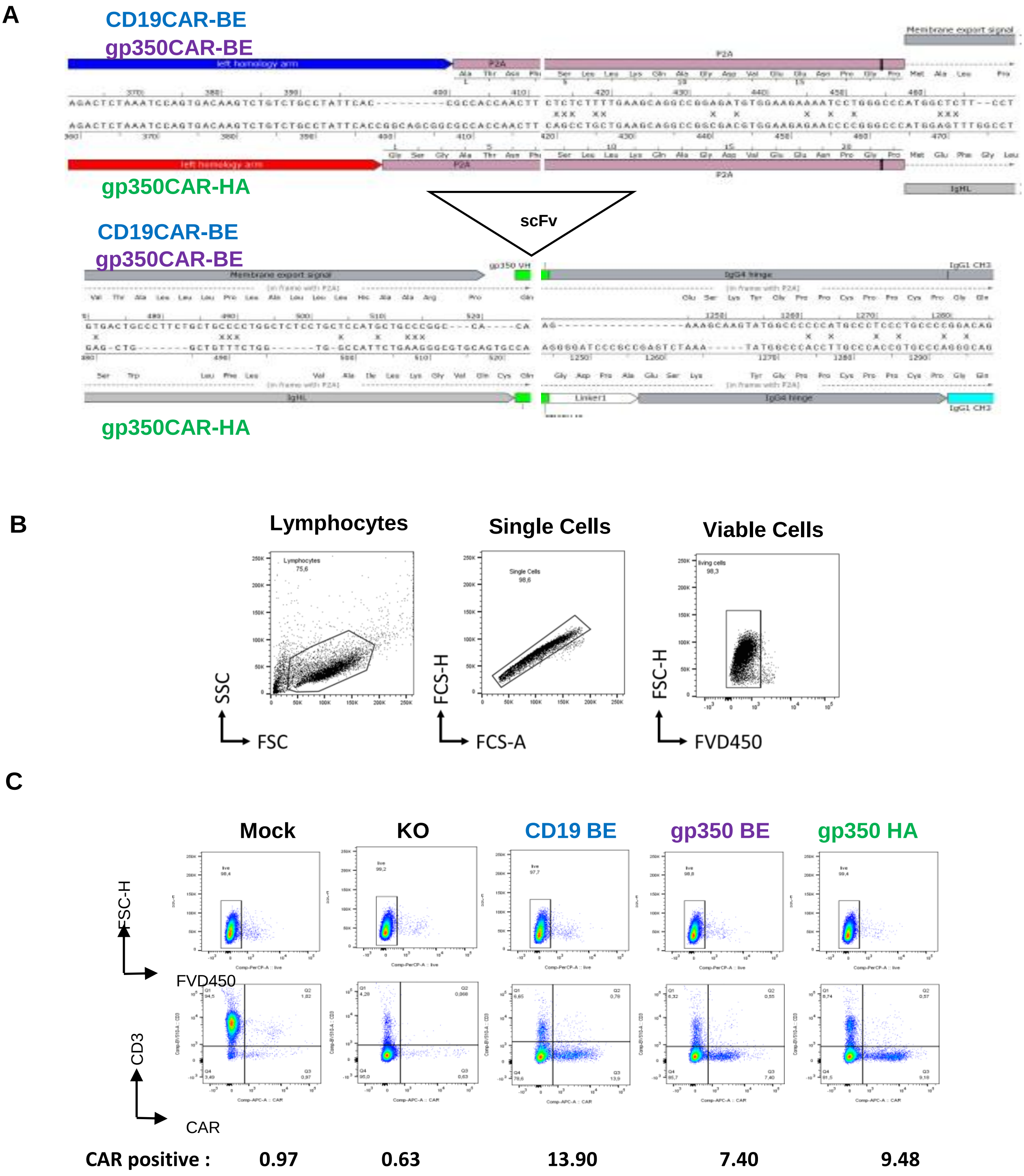

Figure Supplemental 2: FACS analyses of 293T targets and CAR-T effectors after co-culture (related to Figure 2). Gating strategy of 293T/WT or 293T/gp350 cells co-cultured with CD19^{KI} or gp350^{KI}CAR-T cells. First gate on lymphocytes using FSC and SSC. Second gate excluding CD45^{+} cells (CAR-T cells). The 7AAD^{+}CD45^{-} dead target cells were identified.

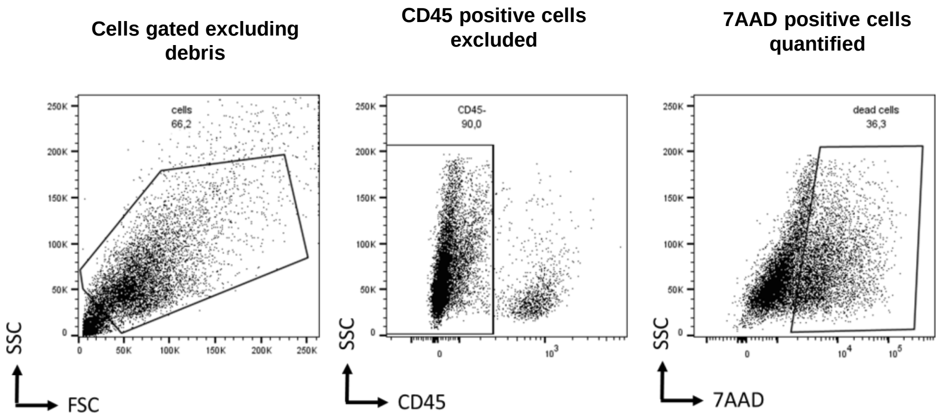

Figure Supplemental 3: FACS analyses and gating strategies of effector CAR-T cells after cryopreservation and thawing and co-cultured with target cells expressing GFP (related to Figures 4). Detection of effector or target cells. First the debris was excluded in FSC and SSC. The viable cells were detected with Fixable viability dye 450. Singlets were gated using FSC-H and FSC-A**.** The target cells were identified as GFP^{+}. Out of the GFP^{-} fraction, the CD4^{+} and CD8^{+} effector cells were identified.

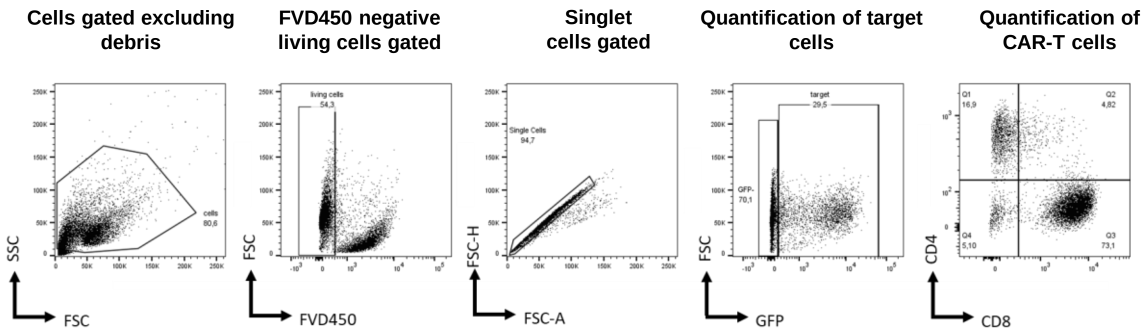

Figure Supplemental 4: FACS analyses and gating strategy to quantify CD19$^+$ (A) or gp350$^+$ (B) cells within lymphoma GFP$^+$ cells that were explanted from tissues of mice challenged with Daudi/GFP-fLuc or Jijoye/GFP-fLuc (related to Figures 5 to 8). Representative examples are shown for bone marrow. Isolated lymphocytes from mice were stained for 30 min in the dark and on ice with or without 1:100 primary 7A1-Ab in PBS. After one wash with 1 % FBS in PBS, the cells were stained again with an Ab-mixture containing anti-CD45 (1:400 pacific blue™ 304022, Biolegend), anti-CD20 (1:100 PE 302306, Biolegend), anti-CD19 (1:100 Alexa Fluor® 700 302225, Biolegend) and Anti-Rat IgG secondary to 7A1 (1:400 Alexa Fluor® 647 AffiniPure Mouse 212-605-082, Jackson Immuno) in PB. The cells were then washed once more in 1% FBS in PBS and then were analyzed in PBS using a LSR II instrument. First gate on lymphocytes using SSC and FSC. Tumor cells were identified as GFP$^+$ Daudi or Jiyoye, and complemented by gating the CD45$^+$ and CD20$^+$ double positive populations in order to reduce background. The frequencies of cells expressing CD19 (A) or gp350 (B) within the tumor cells were then quantified.

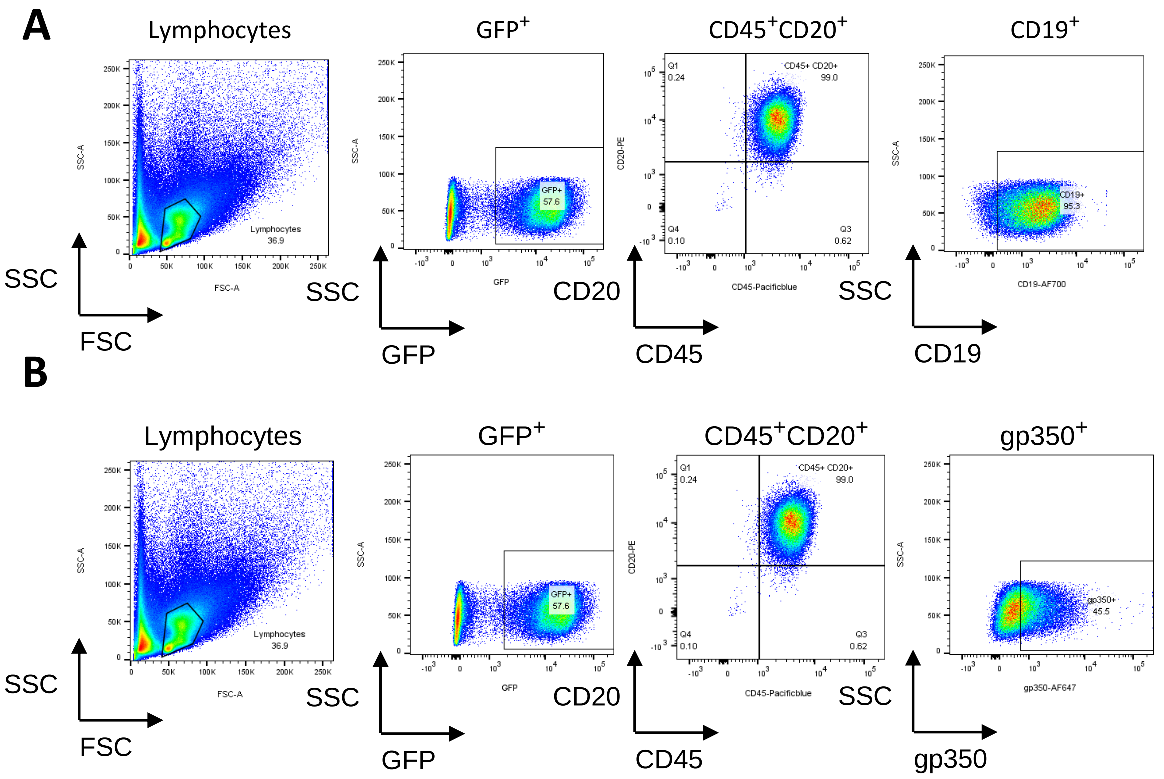

Figure Supplemental 5: (A) FACS analyses and gating strategy to identify and to quantify the frequencies of CAR-T cells in tissues of mice challenged with Jijoye/GFP-fLuc (related to Figures 8H-I). For analysis, the CAR was first stained with Alexa Fluor® 647 AffiniPure F(ab')$_2$ Fragment Goat Anti-Human IgG (109-606-170, Jackson Immuno Research Laboratories. The cells were incubated for 30 min on ice in the dark with 1:100 dilution of the Ab in PBS. Afterwards the cells were washed once with 1% FBS in PBS and then stained in a mixture of anti-CD4 (1:400 PerCP 317432, Biolegend), anti-CD8 (1:200 PE-Cy7 300914, Biolegend) and anti-CD45 (1:400 pacific blue™ 304022, Biolegend) in PBS for 30 min on ice in the dark. After washing in 1% FBS in PBS, the samples were analyzed in PBS using the LSR II instrument. The lymphocytes were gated using FSC and SSC. The CD45^{+}GFP^{-} population was used to identify the human T cells. After gating the CD8^{+} or CD4^{+} cell populations, the frequencies of CAR^{+} cells were determined. (B) Representative examples for gp350^{KI}CAR-T and CD19^{KI}CAR-T cells.

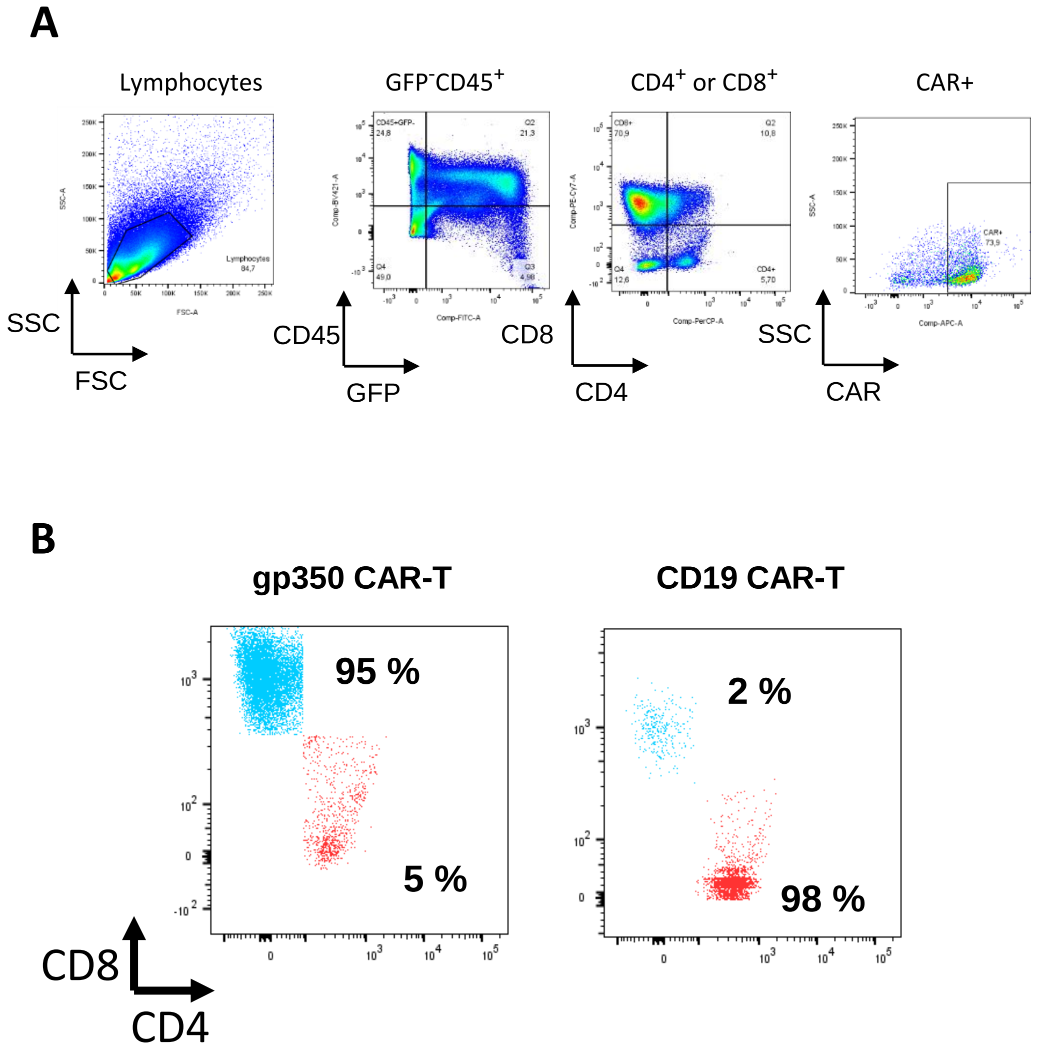

Figure Supplemental 6: FACS analyses and gating strategy for identification of exhaustion markers (related to Figure 9). Representative examples showing a sample of gp350^{KI}CAR-T cells co-cultured with Nalm-6/fLuc-GFP (A) or Jiyoye/fLuc-GFP (B) analyzed after 72h of culture. The IgG receptors were blocked for 10 min on ice with mouse IgG (Sigma Aldrich) and the CAR was stained by incubation with anti-IgG1 antibody for CAR detection (Jackson ImmunoResearch) for 30 minutes at RT. After washing, cells were subsequently stained using the other antibodies (see Supplemental Table 1B) for 30 min at RT. Data was acquired with a MACSQuant® X Flow Cytometer and analyses were done in FlowJo V.10.8 software. Singlets were gated on FSC-A vs. FCS-H and the lymphocytes were gated on the scatter plot (FSC vs. SSC). GFP-positive lymphoma cells were excluded and GFP-negative cells were gated. CAR-positive cells were identified within population. CAR-T cells population were separated into CD4^{+} and CD8^{+} T cells. The expression of the exhaustion markers PD-1, TIM-3 and LAG-3 was determined for each subpopulation.

A **Nalm-6/fLuc-GFP**

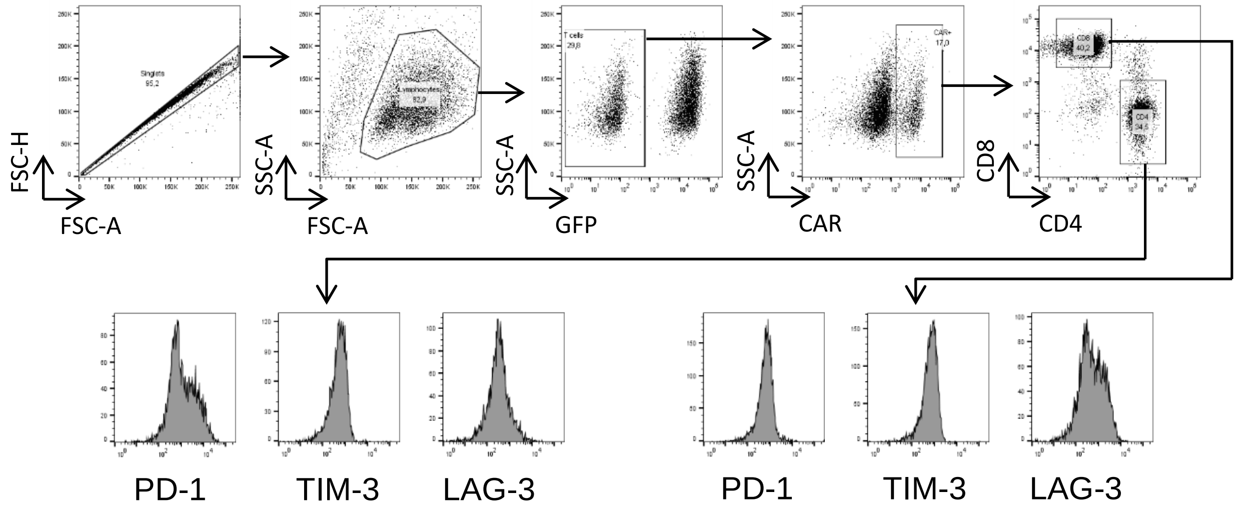

B **Jiyoye/fLuc-GFP**

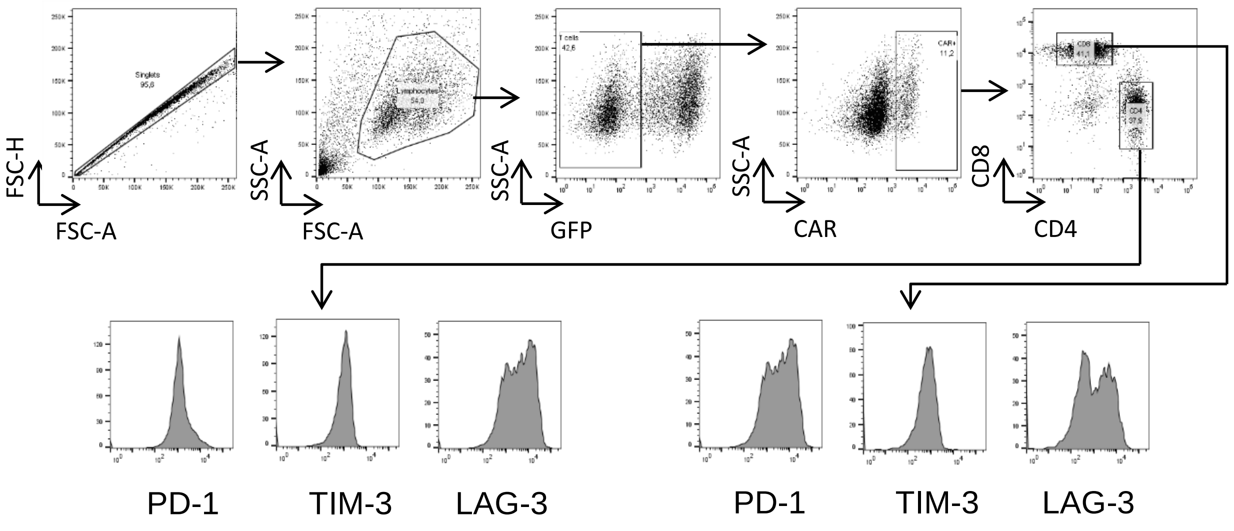

Figure Supplemental 7: Gating strategy for analyses of T_{reg} cells expressing FoxP3 (related to Figure 9). Representative examples showing a sample of a healthy PBMC donor control, $CD19^{KI}$CAR-T cells, $gp350^{KI}$CAR-T cells and bone marrow samples recovered from mice treated with $CD19^{KI}$CAR-T cells or with $gp350^{KI}$CAR-T cells. After FcR blockade (Miltenyi #130-059-901), cells were stained with an antibody against CD4 (see Supplemental Table 1C) for 20 minutes at RT. Subsequently, cells were fixed and permeabilized (Intra Prep, Beckman Coulter #A07803) and intracellular FoxP3 staining was performed. Cells were measured on a MACSQuant® X Flow Cytometer and analyses were done in FlowJo V.10.8 software. Lymphocytes were gated (FSC vs. SSC), and singlets were gated in the FSC-A vs. FSH-H plot. Lymphocytes co-expressing CD4 and $FoxP3^{High}$ cells were identified as T_{regs}.

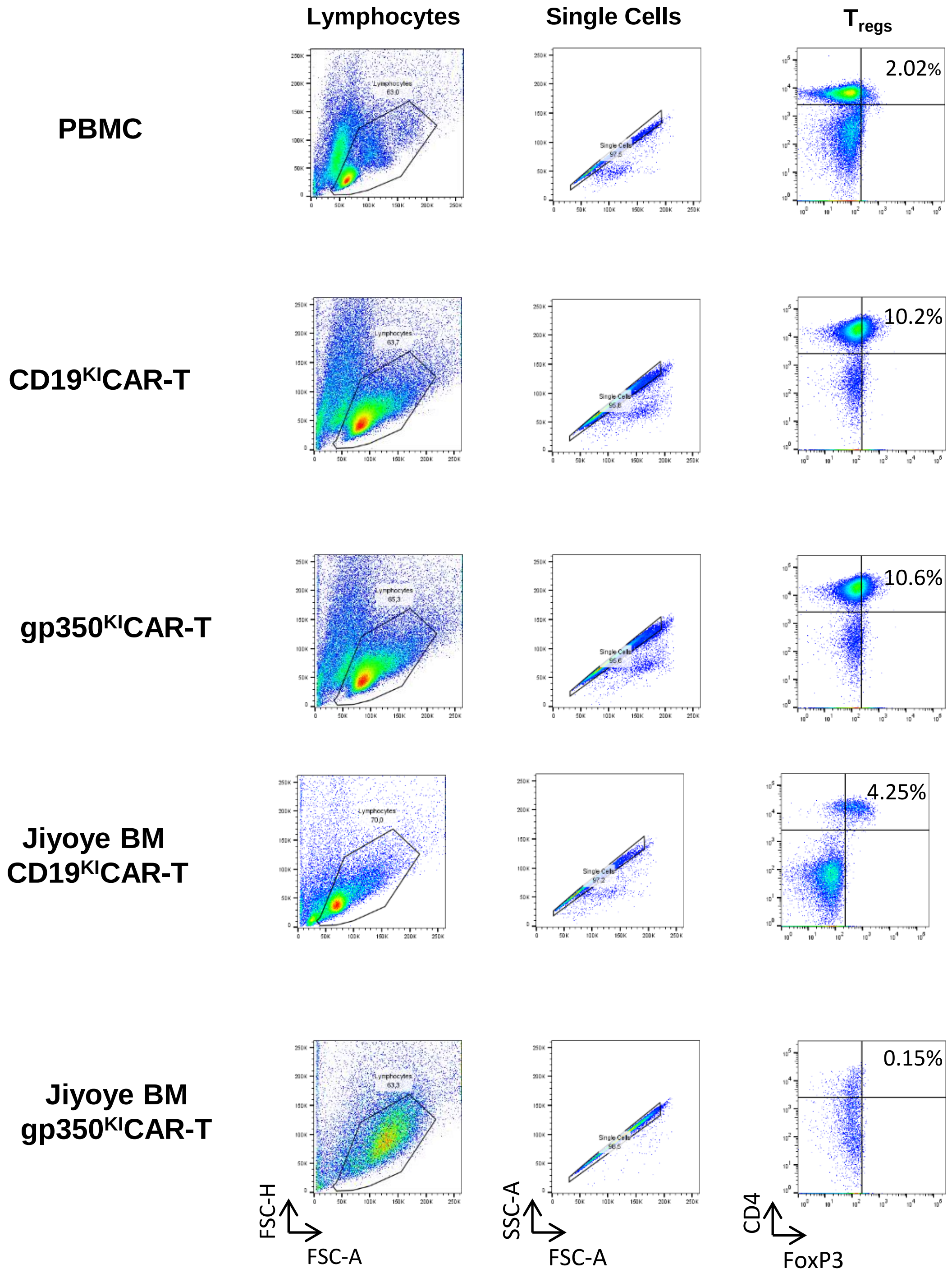

Supplement: Supplementary file 1 [file DataSheet_1.pdf]
